# Macrosomia and Childhood Growth Trajectories From Birth to 10 Years of Age: Findings From the ROLO Longitudinal Birth Cohort Study

**DOI:** 10.1155/jobe/8884369

**Published:** 2025-10-15

**Authors:** Sophie Callanan, Kaat Philippe, Anna Delahunt, Linda M. O'Keeffe, Kate N. O'Neill, Cara A. Yelverton, Catherine M. Phillips, Patrick J. Twomey, Ciara M. McDonnell, Declan Cody, Fionnuala M. McAuliffe

**Affiliations:** ^1^UCD Perinatal Research Centre, School of Medicine, National Maternity Hospital, University College Dublin, Dublin 2, Dublin, Ireland; ^2^School of Public Health, Physiotherapy and Sports Science, University College Dublin, Belfield, Dublin 4, Dublin, Ireland; ^3^School of Public Health, University College Cork, Cork, Ireland; ^4^MRC Integrative Epidemiology Unit, University of Bristol, Bristol, UK; ^5^Population Health Sciences, Bristol Medical School, University of Bristol, Bristol, UK; ^6^Department of Clinical Chemistry, St. Vincent's University Hospital, Dublin, Ireland; ^7^School of Medicine, University College Dublin, Belfield, Dublin 4, Dublin, Ireland; ^8^Department of Paediatric Endocrinology & Diabetes, Children's Health Ireland, Temple Street Hospital, Dublin, Ireland; ^9^Department of Diabetes & Endocrinology, Children's Health Ireland, Crumlin, Dublin, Ireland

## Abstract

**Background/Objective:**

Macrosomia is associated with overweight and obesity across the life course. Most research to date has been based on cross-sectional analyses, and longitudinal investigations between macrosomia and developmental trajectories of growth throughout the first decade of life are lacking. This research aimed to examine associations between macrosomia and postnatal growth trajectories from birth to 10 years of age.

**Subjects:**

Children (*n* = 337) from the ROLO longitudinal birth cohort, who were born to mothers with previous macrosomic delivery.

**Methods:**

Birthweight was recorded at delivery and dichotomised using the cut-off criteria for macrosomia (birthweight ≥ 4 kg and < 4 kg). Child weight, length/height, body mass index (BMI) and waist circumference were measured at birth, 6 months, 2, 5 and 10 years of age. Postnatal growth trajectories were developed using these longitudinal measurements from birth up to 10 years of age. Linear spline multilevel models were used to examine associations between macrosomia and postnatal trajectories with adjustment for confounders (maternal ethnicity, socioeconomic status, maternal age at delivery, maternal smoking in pregnancy, paternal BMI, adherence to gestational weight gain guidelines in pregnancy, sex of the child, original study group allocation, adherence to a special diet in pregnancy, maternal physical activity levels, metabolic complications in pregnancy and breastfeeding).

**Results:**

In this cohort, 53.7% (*n* = 181) had a birthweight ≥ 4 kg. The median (IQR) early pregnancy BMI was 25.4 (23.1, 28.6) kg/m^2^, and mothers were 33.1 (30.6, 35.3) years old at delivery. We found no strong evidence of associations between macrosomia and trajectories of childhood growth from birth to 10 years of age. Significant findings in crude and adjusted models were close to the null and provide limited evidence for a meaningful association.

**Conclusion:**

Macrosomia was associated with early, but not later, childhood growth trajectories. Associations were weak and varied according to definition and growth measurement. The lack of strong results indicates uncertain clinical relevance and warrant additional future research in a larger cohort.

## 1. Introduction

Overweight and obesity (OWOB) is a complex and multifactorial condition that represents a serious public health concern [1]. In 2023, the World Obesity Federation estimated that half of the world's population will have OWOB by 2035 and that childhood and adolescent obesity could more than double compared to 2020 levels [2]. Critical periods for the development of obesity include the prenatal period, the timing of adiposity rebound and the transition to puberty [3]. Understanding risk factors that influence adiposity across these phases of life is important to inform early life obesity interventions.

There is growing interest in the association between high birthweight and postnatal weight status [4]. However, several definitions of high birthweight exist, potentially capturing different genetic and environmental factors driving excess foetal growth. Macrosomia (birthweight ≥ 4 kg or ≥ 4.5 kg) is used to identify neonates with excess weight irrespective of gestational age. Other cut-offs, such as large-for-gestational-age (LGA) (≥ 90^th^ birthweight centile), account for maternal factors, gestational age and infant sex, which may explain above-average growth. Exposure to excess nutrition in utero can stimulate poor glycaemic control and increase fat deposition that continues beyond birth [4]. Children and adolescents born with macrosomia may have a higher risk of OWOB [5–8] and excess fat mass [9–11]. Other research suggests that individuals born with macrosomia have greater subsequent lean mass in later life [12–15]. The majority of research is weakened by cross-sectional analyses of anthropometry that do not capture patterns of growth.

Individuals born with macrosomia may follow distinct growth trajectories which carry unique risks towards the subsequent development of disease in later life. Limited research using trajectory modelling has found that macrosomia is a risk factor for early-onset overweight [16, 17], but most are limited to infancy or early childhood [18, 19]. Rapid weight gain has been associated with higher body mass index (BMI) trajectories throughout childhood and adolescence, with the strongest associations observed among those born with macrosomia [20]. Another study reported that children born with a high birthweight had 2.3 times greater risk of being in a high BMI z-score trajectory group; however, trajectories were only modelled until 4 years of age [21]. Modelled trajectories of growth up to the preteen years in relation to early life factors are scarce [22, 23]. Recent findings reported that high birthweight was a key risk factor for an atypical BMI trajectory at age of 9 years [24]. Additional longitudinal investigations may add relevant information to describe how youth born with macrosomia grow over time. The first decade of life represents a critical period of importance for establishing the age at which these potential associations emerge, due to the evidence that supports the tracking of childhood obesity into adolescence and adulthood [25].

This research aimed to explore the associations between macrosomia and postnatal growth trajectories (weight, length/height, BMI and waist circumference (WC)) from birth to 10 years of age. Data were used from a potentially obesogenic longitudinal birth cohort of children from the Randomised cOntrol trial of a LOw (ROLO) glycaemic index diet in pregnancy to prevent macrosomia study, who were born to mothers who previously delivered an infant with macrosomia. We hypothesise that those born with macrosomia will have different childhood growth trajectories, compared to those born without macrosomia.

## 2. Materials and Methods

### 2.1. Study Design and Population

This is an analysis of the ROLO longitudinal birth cohort study. The primary study was a randomised control trial of a low glycaemic index diet from early pregnancy to prevent the recurrence of macrosomia [26]. Details of the trial protocol, methodology and outcomes have been described previously [26]. The study was carried out between 2007 and 2011 in the National Maternity Hospital, in Dublin, Ireland (registration number ISRCTN54392969). Secundigravida women aged > 18 years, without any pre-existing comorbidities and with a previous history of delivering an infant with macrosomia were eligible for inclusion. Once recruited, the ROLO trial randomised women (*n* = 800) to receive low glycaemic index dietary advice or routine antenatal care (no formal dietary advice) to assess the impact of the intervention on birthweight in a second pregnancy. A total of 759 healthy, singleton infants were born to mothers in the ROLO study. Birthweight did not differ between the trial arms; however, neonates born to mothers in the intervention group had lower thigh skinfolds [27]. The ROLO study continued as a longitudinal birth cohort with follow-up of mother–child dyads at 3 and 6 months postpartum [28, 29], in the ROLO Kids study (at 2 and 5 years of age) [30, 31] and in the ROLO Preteen study (at 9–11 years of age; referred to from this point as 10 years) [12].

### 2.2. Exposure

#### 2.2.1. Macrosomia

At delivery, birthweight was recorded by paediatric medical personnel using a SECA calibrated scales to the nearest 0.1 g. At delivery, the Gestation Network's Bulk Calculator Version 6.2.3 UK was used to calculate birthweight centiles using UK 1990 reference data [32]. To investigate excess weight at birth irrespective of gestational age in relation to long-term postnatal growth, children were dichotomised as those who were born ≥ 4 kg and < 4 kg. Those born with low birthweight or small for gestational age (SGA) were excluded.

### 2.3. Outcomes

#### 2.3.1. Childhood Anthropometry From Birth to 10 Years of Age

Trained research midwives and nutritionists obtained anthropometric measures of weight, length/height, BMI, and WC for each child at multiple time points. Within 2 days of delivery, birth length and WC were measured by the hospital medical staff prior to discharge. At each subsequent time point, WC (at the point of the umbilical scar) was measured to the nearest 0.1 cm using an ergonomic circumference measuring tape (SECA 201 GmbH & co. Kg. Hamburg, Germany). At 6 months, 2, 5 and 10 years of age, weight and length/height were assessed with participants dressed in light clothing and shoes removed. Weight was measured to the nearest 0.1 kg using a digital, stand-on weighing scales (SECA 813 GmbH & co. Kg. Hamburg, Germany) which was calibrated according to the manufacturer's instructions. Height was measured to the nearest 0.1 cm using a free-standing portable stadiometer (SECA 217 GmbH & co. Kg. Hamburg, Germany). Weight and length/height were used to determine BMI as kg/m^2^ for each child starting at birth.

### 2.4. Confounders and Mediators

Confounders and mediators were selected a priori based on previous knowledge of relevant published literature. Maternal ethnicity (White Irish, yes/no) and smoking status during pregnancy (yes/no) were self-reported at the first antenatal visit. A Haase and Pratschke Pobal Index score was determined from each woman's home address as a proxy for socioeconomic status. Total gestational weight gain (GWG) was calculated by subtracting the measured weight at the first antenatal visit from the final weight in pregnancy. Women were categorised as having inadequate/adequate/excessive GWG according to the 2009 Institute of Medicine guidelines [33]. Paternal BMI was self-reported by the mother at the first study visit. The mother's exact age at delivery (years) and sex of the child (male, yes/no) were obtained from hospital medical records. Study group allocation (intervention, yes/no) was also controlled for to account for differences within the sample population. In the first half of pregnancy, questions from Survey of Lifestyle, Attitudes, and Nutrition (SLAN) in Ireland collected self-reported information on typical physical activity levels (calculated as metabolic equivalent units) and whether women were adhering to any of the following diets in pregnancy (irrespective of intervention): vegetarian; vegan; gluten free; low cholesterol; weight reducing; or for diabetes management. Metabolic complications in pregnancy were defined as having one of the following: impaired glucose tolerance at 28 weeks' gestation (one measure of fasting glucose ≥ 5.1 mmol/L, or one measure of glucose > 7.8 mmol/L following the 50g glucose challenge test (GCT); gestational diabetes mellitus (diagnosed when an abnormal GCT was followed by two or more abnormal values on a 3 h 100 g oral glucose tolerance test); or pregnancy-induced hypertension (systolic blood pressure ≥ 140 mmHg and diastolic blood pressure ≥ 90 mmHg on two separate occasions after 20 weeks' gestation) using information from hospital medical records. Data on breastfeeding exposure and duration were obtained retrospectively from postnatal questionnaires at 6 months, 2, 5 and 10 years postnatally.

### 2.5. Participants Eligible for Inclusion

Participants were eligible for inclusion in the current analysis if complete data were obtained for the exposure of interest, complete covariate information available as described above, and at least one measure of weight between birth and 10 years of age, leading to a total sample of 337 children. Of these, fewer children obtained at least one measure of length/height (*n* = 331), BMI (*n* = 331) and WC (*n* = 271).

### 2.6. Statistical Analysis

Statistical analysis was carried out using Stata (SE 18.0). Continuous variables were assessed for normality using Kolmogorov–Smirnov tests and simple histograms. Normally distributed variables were reported as mean and standard deviation (SD), and non-normally distributed variables were reported as median and interquartile range (IQR 25^th^ and 75^th^ percentile). Categorical variables were reported as frequency and percentage *n* (%). Differences in maternal and child characteristics between those born with, compared to without, macrosomia were explored. Independent *t*-test was used to compare normally distributed variables, and Mann–Whitney U test was used to compare non-normally distributed variables between groups. Chi-square tests were used to compare categorical variables.

#### 2.6.1. Linear Spline Multilevel Models

The statistical methods employed in the linear spline multilevel modelling to examine the associations between macrosomia and trajectories of change in weight, length/height, BMI and WC from birth to 10 years of age are described in Supporting Methods. Details of the methodology for the analysis of offspring growth applied in the ROLO cohort have been previously published by O'Keeffe et al. [34]. Four postnatal linear spline periods were used in this analysis from birth–6 months, 6 months–2, 2–5 and 5–10 years.

#### 2.6.2. Associations of Macrosomia With Childhood Growth Trajectories

Separate analyses were performed examining crude and adjusted associations between macrosomia with trajectories of childhood weight, length/height, BMI and WC from birth to 10 years of age. Differences in trajectories in weight, length/height, BMI and WC were explored by including an interaction between macrosomia and the intercept (weight, length/height, BMI and WC at birth) and each spline period. Confounders were included by including an interaction term between each confounder and the intercept and each linear spline period.

### 2.7. Additional Analyses

We examined whether our results were similar when using different definitions including a cut-off value of ≥ 4.5 kg to define extreme cases of macrosomia. Additionally, LGA was characterised as birthweight ≥ 90^th^ and < 90^th^ centile. This clinical cut-off was analysed to account for variability in population characteristics such as maternal weight, maternal height, maternal ethnicity, parity, gestational age at delivery and infant sex. Absolute birthweight was also analysed as a continuous variable (kg) to address loss of statistical power that may have occurred with dichotomisation. We additionally examined the association between macrosomia and weight trajectory from 6 months to 10 years of age. To analyse the effect of the ROLO intervention on the relationship between macrosomia and postnatal growth, the main analyses were stratified and repeated separately for those born to women in the intervention and control groups, respectively.

### 2.8. Sensitivity Analyses

Due to a considerably lower number of participants with available data on breastfeeding (*n* = 183), a sensitivity analysis was performed to additionally explore the possible mediating effect of any breastfeeding in all models (never breastfed/breastfed < 2 months/breastfed ≥ 2 and < 4 months/breastfed ≥ 4 months). Another sensitivity analysis was also performed to repeat the main analysis excluding anthropometric outliers of more than 5 SD from the mean.

## 3. Results

### 3.1. Maternal and Child Characteristics

Cohort characteristics for the 337 mother–child pairs included in this analysis are shown in Table 1. Mothers included in the analysis had a median (IQR 25^th^, 75^th^ percentile) age of 33.1 (30.6, 35.3) years at delivery, and 91.1% were White Irish. The median (IQR 25^th^, 75^th^ percentile) early pregnancy BMI was 25.4 (23.1, 28.6) kg/m^2^, and 46.6% of women experienced excess GWG during their pregnancy. Of 337 infants, the median (IQR 25^th^, 75^th^ percentile) birthweight was 4.02 (3.77, 4.34) kg, and 53.7% (*n* = 181) were born with a birthweight ≥ 4 kg.

When stratified by those born ≥ 4 and < 4 kg, several differences in maternal and child characteristics were found. Differences in maternal and child characteristics stratified by those born ≥ 4.5 and < 4.5 kg, and ≥ 90^th^ centile and < 90^th^ centile are provided in Supporting Tables 1 and 2, respectively. The mean and median child measurements at birth and at each follow-up visit for the total cohort are provided in Supporting Table 3.

### 3.2. Associations Between Macrosomia and Weight Trajectories From Birth to 10 Years of Age

We found no evidence of associations for birthweight ≥ 4 kg compared to < 4 kg and change in weight trajectories from birth to 10 years of age (Table 2 and Supporting Figure 1). Results were similar when analyses were repeated with weight trajectory from 6 months only (Supporting Table 4).

### 3.3. Associations Between Macrosomia and Length/Height Trajectories From Birth to 10 Years of Age

We found no evidence of associations for birthweight ≥ 4 kg compared to < 4 kg and change in length/height trajectories from birth to 10 years of age (Table 3 and Supporting Figure 2).

### 3.4. Associations Between Macrosomia and BMI Trajectories From Birth to 10 Years of Age

The results from crude and adjusted models indicate that birthweight ≥ 4 kg was associated with slower infant growth in BMI trajectory from birth to 6 months of age (−0.03 kg/m^2^/week, 95% CI -0.05, −0.003) (Table 4 and Supporting Figure 3).

### 3.5. Associations Between Macrosomia and WC Trajectories From Birth to 10 Years of Age

The results from crude and adjusted models indicate that birthweight ≥ 4 kg was associated with slower infant growth in WC trajectory from 6 months to 2 years of age (−0.01 cm/week, 95% CI −0.03, −0.0005) (Table 5 and Supporting Figure 4).

### 3.6. Additional Analyses

Results of additional analyses are provided in Supporting Tables 5, 6, 7, and 8. The direction of results from analyses of birthweight ≥ 4.5 kg, birthweight ≥ 90^th^ centile, and continuous birthweight in relation to childhood trajectories from birth to 10 years of age were consistent with our primary analysis. In crude and adjusted models, birthweight ≥ 4.5 kg and birthweight ≥ 90^th^ centile were associated with higher mean measures of weight and length/height at 6 months, 2, 5 and 10 years of age.

### 3.7. Sensitivity Analyses

The main analyses were repeated to additionally adjust for breastfeeding in a subsample of the cohort, and no significant associations were found (Supporting Table 9).

The main analyses were repeated to exclude anthropometric outliers more than 5 SD from the mean for weight (*n* = 1; 17.72 kg at 6 months of age) and BMI (*n* = 1; 39.47 kg/m^2^ at 6 months of age). Results for weight remained similar while the crude and adjusted models indicate that birthweight ≥ 4 kg was associated with faster infant growth in BMI trajectory from 6 months to 2 years of age (0.009 kg/m^2^/week, 95% CI 0.001, 0.01) and slower childhood growth in BMI trajectory from 2 to 5 years of age (−0.003 kg/m^2^/week, 95% CI −0.007, −0.0004) (data are not shown).

Finally, the main analyses were stratified to analyse associations between birthweight ≥ 4 kg and growth trajectories among those born to mothers in the intervention and control group, separately. In the control group only, the crude and adjusted models indicate that birthweight ≥ 4 kg was associated with faster childhood growth in WC trajectory from 2 to 5 years of age (0.01 cm/week, 95% CI 0.002, 0.02), while, in the intervention group, the crude and adjusted models indicate that birthweight ≥ 4 kg was associated with slower childhood growth in WC trajectory from 2 to 5 years of age (−0.01 cm/week, 95% CI −0.02, −0.0007). All other associations in stratified analyses were similar (data not shown).

## 4. Discussion

### 4.1. Main Findings

Size at birth may be associated with the risk of later life OWOB. However, it remains unclear to what extent the risk is inflated by being born with macrosomia [35]. This longitudinal research aimed to contribute to this knowledge gap, by investigating trajectories of growth over a 10-year period in a cohort predisposed to macrosomia. Birthweight ≥ 4 kg was associated with slower infant growth in BMI from birth to 6 months of age and slower infant growth in WC trajectory from 6 months to 2 years of age. However, the magnitude of the associations was small, which provides limited evidence for a clinically meaningful effect. Macrosomia was not associated with any trajectories of childhood growth from 5 to 10 years of age.

### 4.2. Interpretation

Postnatal growth during the first 2 years of life may be a critical developmental period in determining OWOB across the life course for those born at extremes of birth size [36]. Our study found some evidence of slowed BMI growth among those born ≥ 4 kg from birth to 6 months of age, compared to those born < 4 kg. Although weak, our results are consistent with previous studies of growth among larger neonates, who usually experience slowed growth during the first 6 months of life. A small study of 49 neonates found infants born with a higher weight/length ratio or a higher arm-fat area had a slower growth rate until 5 months of age [37]. Another study reported high birthweight infants had slower growth in early infancy, and by 6 months of age, they were anthropometrically similar to normal birthweight infants [38]. These findings are consistent in several other longitudinal studies [39, 40]; however, despite an initial slowdown in growth, infants born with macrosomia continued to remain heavier and taller throughout childhood. While the rationale remains unclear, it is possible that a slower growth increment during the first 6 months of life among infants born with a high birthweight may be due to differences in adaptation to the postnatal environment.

Rapid weight gain before 2 years of age is associated with a higher risk of OWOB in childhood, adolescence and adulthood [35, 41]. Research suggests that a minority of high birthweight infants do not experience adequate catch-down growth in infancy and remain on the upper trajectory of weight gain; however, the desired proportion and optimal timing of growth realignment needs further investigation [42, 43]. However, we observed another period of decelerated growth in WC from 6 months to 2 years of age among those born ≥ 4 kg. Our findings are consistent with those from a small study (*n* = 120) [44] that found those born with macrosomia experienced growth deceleration up to 6 months of age, followed by significant gain during the first 18 months of life, which decreased again gradually during the second and third years of life.

It is also important to consider that the associations found in our analysis, and others, may reflect changes in body composition [35]. There is a paucity of data exploring longitudinal trajectories of body fat in children born with macrosomia. Of the limited available evidence, infants born with macrosomia have greater increases in fat-free mass than fat mass [40]. Another longitudinal study found children born with high birthweight displayed elevated lean mass across the first 2 years of life, while the accumulation of fat mass slowed to a healthier proportion [15]. Evidence suggests differences in body composition may persist into later childhood. In school-aged children, assessment using dual-energy x-ray absorptiometry (DXA) scans revealed those born with high birthweight had higher fat-free mass and similar measures of body fat percentage compared to normal birthweight [45]. Although we found no strong differences in growth patterns, we found that children born with a high birthweight were heavier and taller throughout childhood, compared to their normal birthweight counterparts. These findings may reflect the persistence of taller and leaner stature in childhood, regardless of the rate of growth among those born with macrosomia.

Our analysis found that associations varied according to high birthweight criteria and growth measure, which may have clinical relevance. Macrosomia is more directly tied to birthweight alone, whereas LGA refers to being above average in size for gestational age. Thus, the unique patterns of postnatal growth observed in our analysis may arise due to different determinants (genetic vs. environmental) of their large birth size. Macrosomia and LGA cut-offs capture distinct populations due to a broader range in birthweight among those born LGA, given not all LGA neonates are born with macrosomia. Long-term associations arising from macrosomia may have been more pronounced if we trichotomized the cohort into those born < 4 kg, ≥ 4 to < 4.5 and ≥ 4.5 kg to separate out the degrees of absolute excess weight at birth. Children born ≥ 4 kg to mothers who were randomised to receive low glycaemic index dietary advice had slower WC growth in early childhood, while children born ≥ 4 kg to mothers who received standard care had faster WC growth during the same period. These findings suggest that the intervention may have had a protective effect on abdominal adiposity among children born with a higher birthweight in the preschool years.

Our analysis represents an important contribution to the field that builds on existing research to include trajectories of growth up to the preteen years. This is an important period that includes the adiposity rebound, which refers to the second rise of the BMI curve that usually occurs between 6 and 8 years of age [46]. The timing of the adiposity rebound has been identified as a strong determinant of later life health outcomes including obesity and cardiometabolic diseases [46]. We found no significant associations between macrosomia and trajectories of childhood growth from 5 to 10 years of age. Previous research on whether macrosomia is associated with the timing of the adiposity rebound remains controversial. The majority of research suggests that the risk of early adiposity rebound is higher in those born preterm or SGA [47, 48], with Cissé et al. reporting the average age at adiposity rebound was 91 days earlier in those born SGA compared to LGA [49].

Other considerations for the weak associations found in our analysis should be acknowledged. Maternal factors such as obesity, excess GWG or diabetes may be responsible for in utero programming of postnatal adiposity and metabolism, regardless of size at birth [50, 51]. Previous studies from the ROLO cohort have found that maternal glucose associates with foetal growth trajectories, but maternal metabolic or lipid status does not appear to influence postnatal growth [52–55]. There are also several nonmodifiable genetic drivers of childhood growth such as parental height [56]. It is well recognised that size at birth reflects only one dimension of growth, and the postnatal period may play an equal if not greater role.

### 4.3. Strengths and Limitations

This novel and unique study provides new insights into this understudied and important area. It was well positioned to explore associations of macrosomia with childhood growth, given this cohort was at risk for excess postnatal growth [26]. Analysis of different definitions of high birthweight adds value to this study, given they are rarely investigated simultaneously within the same population. This approach provides a balanced and comprehensive understanding of the relationship between birthweight and subsequent growth, reduces the likelihood of bias from relying on a single cut-off, and increases generalisability to diverse populations and clinical settings globally. Adjusted models included several important maternal and child confounders that all have known associations with foetal or postnatal measures of growth. Linear multilevel modelled splines were chosen for this analysis because of their flexibility and suitability for modelling growth in a longitudinal study [34]. One of the most important advantages is that multilevel models can minimise potential selection bias arising from attrition by including all participants with at least one growth measure under a missing at random assumption. This is particularly useful, despite ongoing efforts to maintain participant engagement in the ROLO birth cohort 10 years later [57]. Linear splines allow growth rates to vary between defined time intervals, enabling a more realistic representation of nonlinear growth patterns without overfitting. Within a multilevel framework, it also accounts for the nonindependence of repeated measures and results in more precise standard errors. Collectively, these strengths made this approach an appropriate and robust choice for modelling trajectories in the ROLO cohort.

Limitations of this research include the small sample size and low statistical power which could explain the lack of strong results and increase the risk of false positive findings. Observed significant findings were close to the null and should be interpreted with caution. There were inconsistent periods between follow-up visits and limited occasions during important growth phases such as catch-down growth and the adiposity rebound. As DXA measures were only obtained at 5 and 10 years of age in the ROLO cohort, additional measures during infancy (from birth to 2 years of age) would have provided greater insight into body composition changes in body fat and lean mass between those born with macrosomia versus without. We used dichotomised variables to analyse the exposures of macrosomia and LGA, which may lose true associations. This approach may have had implications for the specificity of our findings as individuals close to the cut-offs could have been misclassified in terms of risk. In relation to sensitivity, this approach may have grouped those with diverse growth patterns or risk profiles into broad groups. However, to additionally explore a potential dose–response relationship, we supported our findings by analysing birthweight as a continuous variable also. This analysis is also limited by the inability to adjust for other important confounders and mediators such as genetic history (which was not included as an item of interest in the health and lifestyle questionnaires), and fewer participants had available data on breastfeeding which may be independently associated with childhood growth. The novel methods used to develop the growth trajectories in this analysis were of an exploratory design, and they have not been reproduced or validated in other cohorts. It is important to acknowledge that assuming the data were missing at random may introduce bias if missingness is related to unobserved factors such as poorer health status or reduced willingness to participate. Other techniques such as group-based trajectory modelling or latent class growth modelling were not chosen because they are more complex, difficult to interpret, and are heavily dependent on the assumption that there are distinct groups within the data. However, our findings and earlier results in the same cohort suggest that our modelling approach may be limited in its ability to identify differences of clinical and public health relevance.

### 4.4. Recommendations for Future Research

Our findings have uncertain clinical relevance, and future research is crucial to disentangle the associations between size at birth, postnatal growth and future risk of obesity. Evidence in this area would be strengthened through the investigation of the rate of fat and fat-free mass accretion and novel biomarkers as indices of adipose tissue function, beginning at birth [41]. Given the lack of strong results in this cohort using multilevel linear spline models, future work may explore the appropriateness of alternative statistical methods that do not rely on the assumption of a single average population trajectory. Additionally, exploration of nonlinear growth patterns such as fractional polynomials may provide valuable insights on the timing of the adiposity rebound in relation to birthweight and postnatal growth, in which this study did not capture because we only had one measure available at 5 and 10 years of age. Finally, additional work in larger populations of youth with more frequent measurements is important to build on these findings, before we can ascertain if there is a longitudinal association between size at birth and childhood growth and adiposity.

### 4.5. Clinical and Public Health Policy Implications

Primary prevention strategies that educate and support pregnant women to maintain healthy weight and glycaemic control throughout their pregnancies may be beneficial to ensure offspring establish a healthy weight trajectory in utero [58], that is, sustained throughout childhood and later life [21]. Hopeful secondary prevention strategies in early life for decreasing the risk of obesity among those born with macrosomia include the promotion of breastfeeding, appropriate timing for the introduction of solids, along with healthy diet and physical activity habits throughout childhood [21, 41, 59, 60]. Despite our weak findings, the broader literature suggests that infants born with macrosomia may benefit from long-term surveillance of growth patterns in childhood to identify opportunities for early obesity prevention and management [21, 41, 44].

## 5. Conclusion

This study found some associations between macrosomia and early growth trajectories, such as birthweight ≥ 4 kg was associated with slower infant growth in BMI from birth to 6 months of age and slower infant growth in WC trajectory from 6 months to 2 years of age. The magnitude of the results was small, and associations varied according to macrosomia criteria and growth measure, indicating that they have uncertain clinical relevance. Future research may benefit from modelling trajectories of body composition in a larger cohort of youth born with macrosomia, to better ascertain the link between size at birth and risk of OWOB in later life.

## Figures and Tables

**Table 1:** Maternal and child characteristics from the ROLO study and differences between those born ≥ 4 kg compared to < 4 kg.

	Total		Birthweight ≥ 4 kg		Birthweight < 4 kg		*p*
	*N*	Mean (SD)/median (IQR)/*n* (%)	*N*	Mean (SD)/median (IQR)/*n* (%)	*N*	Mean (SD)/median (IQR)/*n* (%)	
Age at delivery (years)	337	33.1 (30.6, 35.3)	181	33.2 (30.1, 35.1)	156	32.9 (31.2, 35.4)	0.77
HP index	337	7.0 (−0.9, 11.8)	181	7.0 (−0.8, 11.4)	156	7.2 (−2.10, 12.4)	0.86
Early pregnancy BMI (kg/m^2^)	337	25.4 (23.1, 28.6)	181	25.6 (23.4, 29.3)	156	25.3 (22.7, 27.6)	0.09
Paternal BMI (kg/m^2^)	337	27.2 (25.1, 29.4)	181	27.0 (25.0, 29.2)	156	27.7 (25.4, 29.6)	0.24
Ethnicity							
White Irish, *n* (%)	337	307 (91.1)	181	167 (92.3)	156	140 (89.7)	0.41
Other, *n* (%)	337	30 (8.9)	181	14 (7.7)	156	16 (10.3)	
Gestational weight gain							
Total (kg)	337	13.4 (4.39)	181	13.9 (4.5)	156	12.8 (4.2)	0.021
Inadequate, *n* (%)	337	48 (14.2)	181	17 (9.4)	156	31 (19.9)	0.012
Adequate, *n* (%)	337	132 (39.2)	181	70 (38.7)	156	62 (39.7)	
Excessive, *n* (%)	337	157 (46.6)	181	94 (51.9)	156	63 (40.4)	
Metabolic complication in pregnancy							
Yes, *n* (%)	337	94 (27.9)	181	41 (22.7)	156	53 (34.0)	0.021
No, *n* (%)	337	243 (72.1)	181	140 (77.3)	156	103 (66.0)	
Study group allocation							
Intervention, *n* (%)	337	175 (51.9)	181	96 (53.0)	156	79 (50.6)	0.66
Control, *n* (%)	337	162 (48.1)	181	85 (47.0)	156	77 (49.4)	
Smoking in pregnancy							
Yes, *n* (%)	337	14 (4.2)	181	7 (3.9)	156	7 (4.5)	0.77
No, *n* (%)	337	323 (95.8)	181	174 (96.1)	156	149 (95.5)	
Adherence to a special diet in pregnancy							
Yes, *n* (%)	337	57 (16.9)	181	30 (16.6)	156	27 (17.3)	0.85
No, *n* (%)	337	280 (83.1)	181	151 (83.4)	156	129 (82.7)	
Physical activity level (METs/day)	337	330.0 (198.0, 551.0)	181	358.0 (198.0, 560.0)	156	325.0 (198.0, 504.0)	0.35
Child sex							
Male, *n* (%)	337	158 (46.9)	181	102 (56.4)	156	56 (35.9)	< 0.001
Female, *n* (%)	337	179 (53.1)	181	79 (43.6)	156	100 (64.1)	
Exposure to breast milk							
Never, *n* (%)	183	70 (38.3)	96	40 (41.7)	87	30 (34.5)	0.71
< 2 months, *n* (%)	183	18 (9.8)	96	8 (8.3)	87	10 (11.5)	
≥ 2 and < 4 months, *n* (%)	183	26 (14.2)	96	14 (14.6)	87	12 (13.8)	
≥ 4 months, *n* (%)	183	69 (37.7)	96	34 (18.8)	87	30 (34.5)	
Birthweight (kg)	337	4.02 (3.77, 4.34)	181	4.31 (4.14, 4.55)	156	3.75 (3.53, 3.87)	< 0.001
Birthweight centile	337	80.5 (60.9, 93.8)	181	91.8 (79.9, 97.1)	156	62.5 (41.5, 79.2)	< 0.001
GA at delivery (days)	337	283.0 (277.5, 288.0)	181	285.0 (280.0, 290.0)	156	280.0 (274.0, 285.0)	< 0.001

*Note:* Randomised cOntrol trial of a LOw glycaemic index diet in pregnancy to prevent macrosomia; HP Haase and Pratschke Deprivation index. Results presented as mean (SD) for normally distributed variables, median (IQR 25^th^, 75^th^ percentile) for non-normally distributed variables, or frequency and percentage *n* (%) for categorical variables. *p*-values determined using independent *t*-tests for normally distributed variables, Mann–Whitney U tests for non-normally distributed variables, or Chi-square tests for categorical variables; *p* < 0.05 considered statistically significant. *N* = total population; *n* = frequency.

Abbreviations: BMI, body mass index; GA, gestational age; IQR, interquartile range; METs, metabolic equivalent units; SD, standard deviation.

**Table 2:** Crude and adjusted mean trajectory and mean difference in trajectory of weight from birth to 10 years of age for birthweight ≥ 4 kg, estimated from multilevel linear spline models.

	Mean weight trajectory (95% CI) (kg) for those born < 4 kg	Difference (95% CI) in mean weight trajectory for those born ≥ 4 kg
*Unadjusted*		
Weight at birth (kg)	3.75 (3.71, 3.80)	0.55 (0.49, 0.62)
Δ birth–6 months (kg/week)	0.15 (0.14, 0.17)	0.005 (−0.009, 0.02)
Weight at 6 months (kg)	7.91 (7.63, 8.20)	0.71 (0.32, 1.10)
Δ 6 months–2 years (kg/week)	0.05 (0.05, 0.06)	0.0001 (−0.006, 0.006)
Weight at 2 years (kg)	12.4 (12.04, 12.8)	0.72 (0.17, 1.27)
Δ 2 years–5 years (kg/week)	0.04 (0.04, 0.05)	−0.0006 (−0.003, 0.002)
Weight at 5 years (kg)	19.3 (18.8, 19.8)	0.62 (−0.02, 1.26)
Δ 5 years–10 years (kg/week)	0.06 (0.06, 0.07)	−0.001 (−0.009, 0.005)
Weight at 10 years (kg)	36.5 (35.1, 37.9)	0.13 (−1.80, 2.06)

*Adjusted*		
Weight at birth (kg)	3.62 (3.50, 3.74)	0.54 (0.48, 0.61)
Δ birth–6 months (kg/week)	0.13 (0.10, 0.17)	−0.0008 (−0.01, 0.01)
Weight at 6 months (kg)	7.21 (6.30, 8.12)	0.52 (0.11, 0.93)
Δ 6 months–2 years (kg/week)	0.06 (0.04, 0.08)	0.001 (−0.005, 0.008)
Weight at 2 years (kg)	12.3 (11.1, 13.5)	0.65 (0.08, 1.21)
Δ 2 years–5 years (kg/week)	0.04 (0.03, 0.05)	−0.001 (−0.004, 0.001)
Weight at 5 years (kg)	19.4 (18.1, 20.7)	0.44 (−0.20, 1.09)
Δ 5 years–10 years (kg/week)	0.07 (0.05, 0.08)	−0.001 (−0.004, 0.001)
Weight at 10 years (kg)	37.8 (34.2, 41.4)	0.01 (−1.86, 1.89)

*Note:* Δ the change. Adjusted models controlled for original study group, child sex, HP index, maternal age at delivery, maternal ethnicity, gestational weight gain, maternal smoking in pregnancy, maternal physical activity in pregnancy, adherence to a special diet in pregnancy, metabolic complications in pregnancy, and paternal BMI. Mean trajectory is centred on the mean of the first measurement (birth) as the reference category. *N* = 337.

Abbreviation: CI, confidence interval.

**Table 3:** Crude and adjusted mean trajectory and mean difference in trajectory of length/height from birth to 10 years of age for birthweight ≥ 4 kg, estimated from multilevel linear spline models.

	Mean length/height trajectory (95% CI) (cm) for those born < 4 kg	Difference (95% CI) in mean length/height trajectory for those born ≥ 4 kg
*Unadjusted*		
Length at birth (cm)	52.2 (51.9, 52.5)	1.37 (0.97, 1.77)
Δ birth–6 months (cm/week)	0.63 (0.61, 0.66)	0.01 (−0.02, 0.04)
Length at 6 months (cm)	68.8 (68.2, 69.5)	1.66 (0.78, 2.54)
Δ 6 months–2 years (cm/week)	0.25 (0.24, 0.26)	−0.007 (−0.02, 0.005)
Height at 2 years (cm)	88.5 (87.8, 89.2)	1.06 (0.08, 2.05)
Δ 2 years–5 years (cm/week)	0.13 (0.13, 0.14)	−0.0007 (−0.007, 0.005)
Height at 5 years (cm)	109.6 (108.8, 110.4)	0.94 (−0.18, 2.07)
Δ 5 years–10 years (cm/week)	0.12 (0.11, 0.12)	−0.0007 (−0.004, 0.003)
Height at 10 years (cm)	141.1 (140.1, 142.1)	0.74 (−0.62, 2.11)

*Adjusted*		
Length at birth (cm)	52.3 (51.5, 53.1)	1.23 (0.83, 1.63)
Δ birth–6 months (cm/week)	0.57 (0.50, 0.65)	−0.01 (−0.04, 0.02)
Length at 6 months (cm)	67.3 (65.3, 69.3)	0.88 (−0.01, 1.78)
Δ 6 months–2 years (cm/week)	0.25 (0.22, 0.28)	−0.004 (−0.01, 0.009)
Height at 2 years (cm)	87.1 (85.1, 89.2)	0.53 (−0.44, 1.51)
Δ 2 years–5 years (cm/week)	0.14 (0.13, 0.16)	−0.0004 (−0.007, 0.006)
Height at 5 years (cm)	109.9 (107.7, 112.1)	0.46 (−0.66, 1.60)
Δ 5 years–10 years (cm/week)	0.12 (0.11, 0.13)	−0.00005 (−0.004, 0.004)
Height at 10 years (cm)	142.1 (139.4, 144.7)	0.45 (−0.90, 1.81)

*Note:* Δ the change. Adjusted models controlled for original study group, child sex, HP index, maternal age at delivery, maternal ethnicity, gestational weight gain, maternal smoking in pregnancy, maternal physical activity in pregnancy, adherence to a special diet in pregnancy, metabolic complications in pregnancy, and paternal BMI. Mean trajectory is centred on the mean of the first measurement (birth) as the reference category. *N* = 331.

Abbreviation: CI, confidence interval.

**Table 4:** Crude and adjusted mean trajectory and mean difference in trajectory of BMI from birth to 10 years of age for birthweight ≥ 4 kg, estimated from multilevel linear spline models.

	Mean BMI trajectory (95% CI) (kg/m^2^) for those born < 4 kg	Difference (95% CI) in mean BMI trajectory for those born ≥ 4 kg
*Unadjusted*		
BMI at birth (kg/m^2^)	13.7 (13.5, 13.9)	1.27 (0.98, 1.56)
Δ birth–6 months (kg/m^2^/week)	0.12 (0.10, 0.14)	−0.02 (−0.05, −0.002)
BMI at 6 months (kg/m^2^)	16.9 (16.5, 17.4)	0.54 (−0.05, 1.13)
Δ 6 months–2 years (kg/m^2^/week)	−0.01 (−0.02, −0.01)	0.001 (−0.007, 0.01)
BMI at 2 years (kg/m^2^)	15.6 (15.2, 16.1)	0.67 (0.09, 1.25)
Δ 2 years–5 years (kg/m^2^/week)	0.001 (−0.001, 0.005)	−0.003 (−0.007, 0.0008)
BMI at 5 years (kg/m^2^)	15.9 (15.5, 16.3)	0.16 (−0.33, 0.67)
Δ 5 years–10 years (kg/m^2^/week)	0.007 (0.005, 0.01)	−0.0006 (−0.003, 0.002)
BMI at 10 years (kg/m^2^)	17.9 (17.4, 18.5)	0.0007 (−0.77, 0.78)

*Adjusted*		
BMI at birth (kg/m^2^)	13.2 (12.7, 13.7)	1.31 (1.01, 1.60)
Δ birth–6 months (kg/m^2^/week)	0.12 (0.06, 0.18)	−0.03 (−0.05, −0.003)
BMI at 6 months (kg/m^2^)	16.5 (15.1, 18.0)	0.51 (−0.11, 1.14)
Δ 6 months–2 years (kg/m^2^/week)	−0.008 (−0.03, 0.01)	0.002 (−0.007, 0.01)
BMI at 2 years (kg/m^2^)	15.9 (14.6, 17.2)	0.74 (0.13, 1.34)
Δ 2 years–5 years (kg/m^2^/week)	0.0009 (−0.008, 0.01)	−0.003 (−0.007, 0.0005)
BMI at 5 years (kg/m^2^)	16.1 (15.1, 17.1)	0.16 (−0.37, 0.69)
Δ 5 years–10 years (kg/m^2^/week)	0.008 (0.002, 0.01)	−0.0006 (−0.003, 0.002)
BMI at 10 years (kg/m^2^)	18.3 (16.8, 19.8)	−0.01 (−0.76, 0.73)

*Note:* Δ the change. Adjusted models controlled for original study group, child sex, HP index, maternal age at delivery, maternal ethnicity, gestational weight gain, maternal smoking in pregnancy, maternal physical activity in pregnancy, adherence to a special diet in pregnancy, metabolic complications in pregnancy, and paternal BMI. Mean trajectory is centred on the mean of the first measurement (birth) as the reference category. *N* = 331.

Abbreviations: BMI, body mass index; CI, confidence interval.

**Table 5:** Crude and adjusted mean trajectory and mean difference in trajectory of WC from birth to 10 years of age for birthweight ≥ 4 kg, estimated from multilevel linear spline models.

	Mean WC (95% CI) (cm) for those born < 4 kg	Difference (95% CI) in mean WC trajectory for those born ≥ 4 kg
*Unadjusted*		
WC at birth (cm)	32.1 (31.3, 32.9)	2.51 (1.49, 3.53)
Δ birth–6 months (cm/week)	0.38 (0.33, 0.42)	−0.01 (−0.07, 0.04)
WC at 6 months (cm)	42.1 (41.2, 42.9)	2.14 (1.00, 3.28)
Δ 6 months–2 years (cm/week)	0.09 (0.08, 0.11)	−0.02 (−0.04, −0.005)
WC at 2 years (cm)	49.7 (48.9, 50.5)	0.31 (−0.77, 1.40)
Δ 2 years–5 years (cm/week)	0.03 (0.02, 0.03)	−0.0005 (−0.009, 0.008)
WC at 5 years (cm)	54.7 (53.9, 55.5)	0.23 (−0.89, 1.35)
Δ 5 years–10 years (cm/week)	0.03 (0.03, 0.04)	−0.003 (−0.01, 0.005)
WC at 10 years (cm)	64.9 (63.1, 66.7)	−0.74 (−3.23, 1.73)

*Adjusted*		
WC at birth (cm)	32.7 (30.8, 34.5)	2.83 (1.76, 3.90)
Δ birth–6 months (cm/week)	0.34 (0.21, 0.46)	−0.03 (−0.09, 0.02)
WC at 6 months (cm)	41.6 (38.9, 44.2)	1.86 (0.69, 3.04)
Δ 6 months–2 years (cm/week)	0.10 (0.06, 0.14)	−0.01 (−0.03, −0.0005)
WC at 2 years (cm)	49.7 (47.2, 52.1)	0.38 (−0.74, 1.50)
Δ 2 years–5 years (cm/week)	0.03 (0.01, 0.05)	−0.001 (−0.009, 0.007)
WC at 5 years (cm)	54.8 (52.5, 57.0)	0.20 (−0.93, 1.34)
Δ 5 years–10 years (cm/week)	0.03 (0.02, 0.05)	−0.004 (−0.01, 0.005)
WC at 10 years (cm)	64.7 (60.1, 69.4)	−0.83 (−3.24, 1.56)

*Note:* Δ the change. Adjusted models controlled for original study group, child sex, HP index, maternal age at delivery, maternal ethnicity, gestational weight gain, maternal smoking in pregnancy, maternal physical activity in pregnancy, adherence to a special diet in pregnancy, metabolic complications in pregnancy, and paternal BMI. Mean trajectory is centred on the mean of the first measurement (birth) as the reference category. *N* = 271.

Abbreviations: CI, confidence interval; WC, waist circumference.

## Data Availability

The data that support the findings of this study are available from the corresponding author upon reasonable request.

## Nomenclature

BMI: Body mass index
DXA: Dual-energy x-ray absorptiometry
LGA: Large-for-gestational-age
GWG: Gestational weight gain
OWOB: Overweight and obesity
ROLO: Randomised cOntrol trial of a LOw glycaemic index diet in pregnancy to prevent macrosomia
SGA: Small-for-gestational-age
WC: Waist circumference

## Supporting Information

Supporting Methods: Modelling of trajectories.

Supporting Figure 1: Weight trajectories from birth to 10 years of age for those born with and without macrosomia (birthweight ≥ 4 kg).

Supporting Figure 2: Length/height trajectories from birth to 10 years of age for those born with and without macrosomia (birthweight ≥ 4 kg).

Supporting Figure 3: BMI trajectories from birth to 10 years of age for those born with and without macrosomia (birthweight ≥ 4 kg).

Supporting Figure 4: Waist circumference trajectories from birth to 10 years of age for those born with and without macrosomia (birthweight ≥ 4 kg).

Supporting Table 1: Differences in maternal and child characteristics between those born ≥ 4.5 kg compared to < 4.5 kg.

Supporting Table 2: Differences in maternal and child characteristics between those born ≥ 90^th^ centile compared to < 90^th^ centile.

Supporting Table 3: Anthropometric characteristics from birth to 10 years of age.

Supporting Table 4: Crude and adjusted mean trajectory and mean difference in trajectory of weight from 6 months to 10 years of age for birthweight ≥ 4 kg, estimated from multilevel linear spline models.

Supporting Table 5: Crude and adjusted mean trajectory and mean difference in trajectory of WC from birth to 10 years of age for birthweight (≥ 4.5 kg; ≥ 90^th^ centile; continuous), estimated from multilevel linear spline models.

Supporting Table 6: Crude and adjusted mean trajectory and mean difference in trajectory of weight from birth to 10 years of age for birthweight (≥ 4.5 kg; ≥ 90^th^ centile; continuous), estimated from multilevel linear spline models.

Supporting Table 7: Crude and adjusted mean trajectory and mean difference in trajectory of length/height from birth to 10 years of age for birthweight (≥ 4.5 kg; ≥ 90^th^ centile; continuous), estimated from multilevel linear spline models.

Supporting Table 8: Crude and adjusted mean trajectory and mean difference in trajectory of BMI from birth to 10 years of age for birthweight (≥ 4.5 kg; ≥ 90^th^ centile; continuous), estimated from multilevel linear spline models.

Supporting Table 9: Sensitivity analyses of adjusted mean trajectory and mean difference in trajectory of anthropometry from birth to 10 years of age for birthweight ≥ 4 kg additionally adjusting for breastfeeding, estimated from multilevel linear spline models.
