# Supplementary material for: Macrosomia and Childhood Growth Trajectories From Birth to 10 Years of Age: Findings From the ROLO Longitudinal Birth Cohort Study
Source: J Obes. 2025 Oct 15;2025:8884369. doi: 10.1155/jobe/8884369 (PMC12543617; doi:10.1155/jobe/8884369)
Supplement: Supporting Information — Additional supporting information can be found online in the Supporting Information section. [file 8884369.f1.docx]

**Macrosomia and childhood growth trajectories from birth to 10 years of age: findings from the ROLO longitudinal birth cohort study**

**Supplementary Material**

**Authors:** Sophie Callanan^1^, Kaat Philippe^2^, Anna Delahunt^1^, Linda M O’Keeffe^3,4,5^, Kate N O’Neill^3^, Cara A Yelverton^1^, Catherine M Phillips^2^, Patrick J Twomey^6,7^, Ciara M McDonnell^8^, Declan Cody^9^, Fionnuala M McAuliffe^1^

**Corresponding author:** Professor Fionnuala M McAuliffe, UCD Perinatal Research Centre, School of Medicine, University College Dublin, The National Maternity Hospital, Dublin 2, Ireland, Telephone: +353 1 637 3216, Fax: +353 1 662 7586, E-mail: [fionnuala.mcauliffe@ucd.ie](mailto:fionnuala.mcauliffe@ucd.ie)

**Supplementary Methods**

*Modelling of trajectories*

Linear spline multilevel models were used to estimate trajectories of change for growth measurements including weight, length/height, BMI and WC from birth to 10 years of age. Multilevel models estimate mean trajectories of the outcome of interest, while accounting for non-independence (that is clustering) of repeated measurements within individuals, change in scale and variance of measures over time and differences in the number and timing of measurements between individuals (using of all available data from all eligible study participants under a missing at random assumption (1, 2)). This approach enables us to include participants, regardless of whether they had 1 or multiple measures of growth across the period of follow up. The multilevel models consist of two levels that include measurement occasion and individual. Linear splines allow knot plots to be fit at different ages to derive periods in which change is approximately linear. The optimal linear spline model for each growth measure was selected by examining observed data for each growth measure and comparing model fit statistics for different models. Strategies for selection of knot points are described in further detail elsewhere (1). The best fitting model for each anthropometric measure included a model with knots at each measurement occasion giving rise to three postnatal linear spline periods: from birth – 6 months, 6 months – 2 years, and 2 years – 5 years (3). For the current analysis, the model was extended to include a fourth postnatal linear spline period from 5 years – 10 years. It was not possible to explore or model non-linear change to account for the adiposity rebound between 5 and 10 years of age due to the limited data available. The trajectories were centred on the mean of the first available measure (at birth for weight, length/height, BMI and WC). No restrictions were placed on the variance – covariance matrices of level 2 (individual level) random effects for all models.

**References**

1. Howe LD, Tilling K, Matijasevich A, Petherick ES, Santos AC, Fairley L, et al. Linear spline multilevel models for summarising childhood growth trajectories: a guide to their application using examples from five birth cohorts. Statistical methods in medical research. 2016;25(5):1854-74.

2. Tilling K, Macdonald-Wallis C, Lawlor DA, Hughes RA, Howe LD. Modelling childhood growth using fractional polynomials and linear splines. Annals of Nutrition and Metabolism. 2014;65(2-3):129-38.

3. O'Keeffe LM, Yelverton CA, Bartels HC, O'Neill KN, McDonnell C, McAuliffe FM. Application of multilevel linear spline models for analysis of growth trajectories in a cohort with repeat antenatal and postnatal measures of growth: a prospective cohort study. BMJ open. 2023;13(3):e065701.

**Supplementary Figures**

**
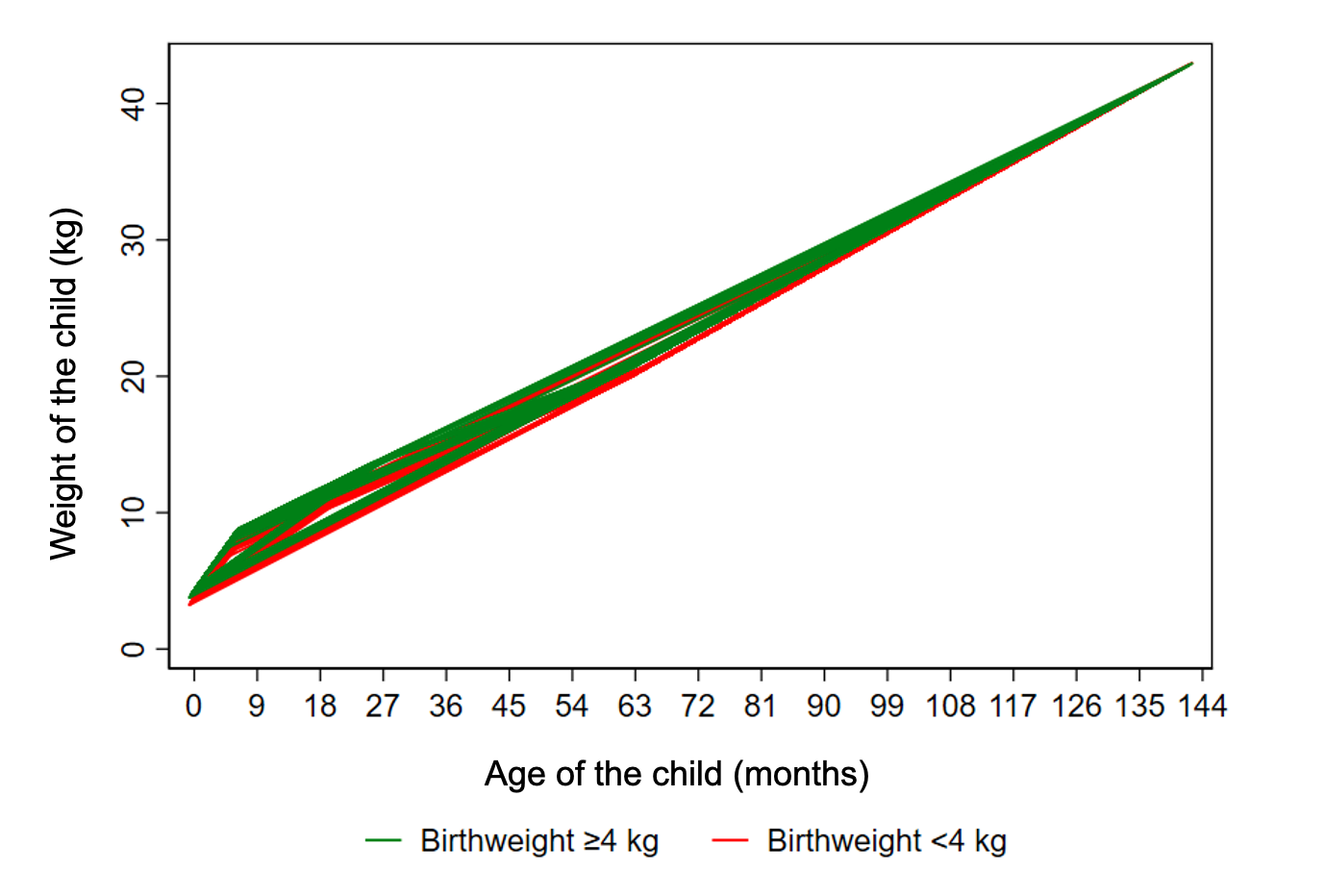
**

**Supplementary Figure 1:** Weight trajectories from birth to 10 years of age for those born with and without macrosomia (birthweight ≥4 kg).

**
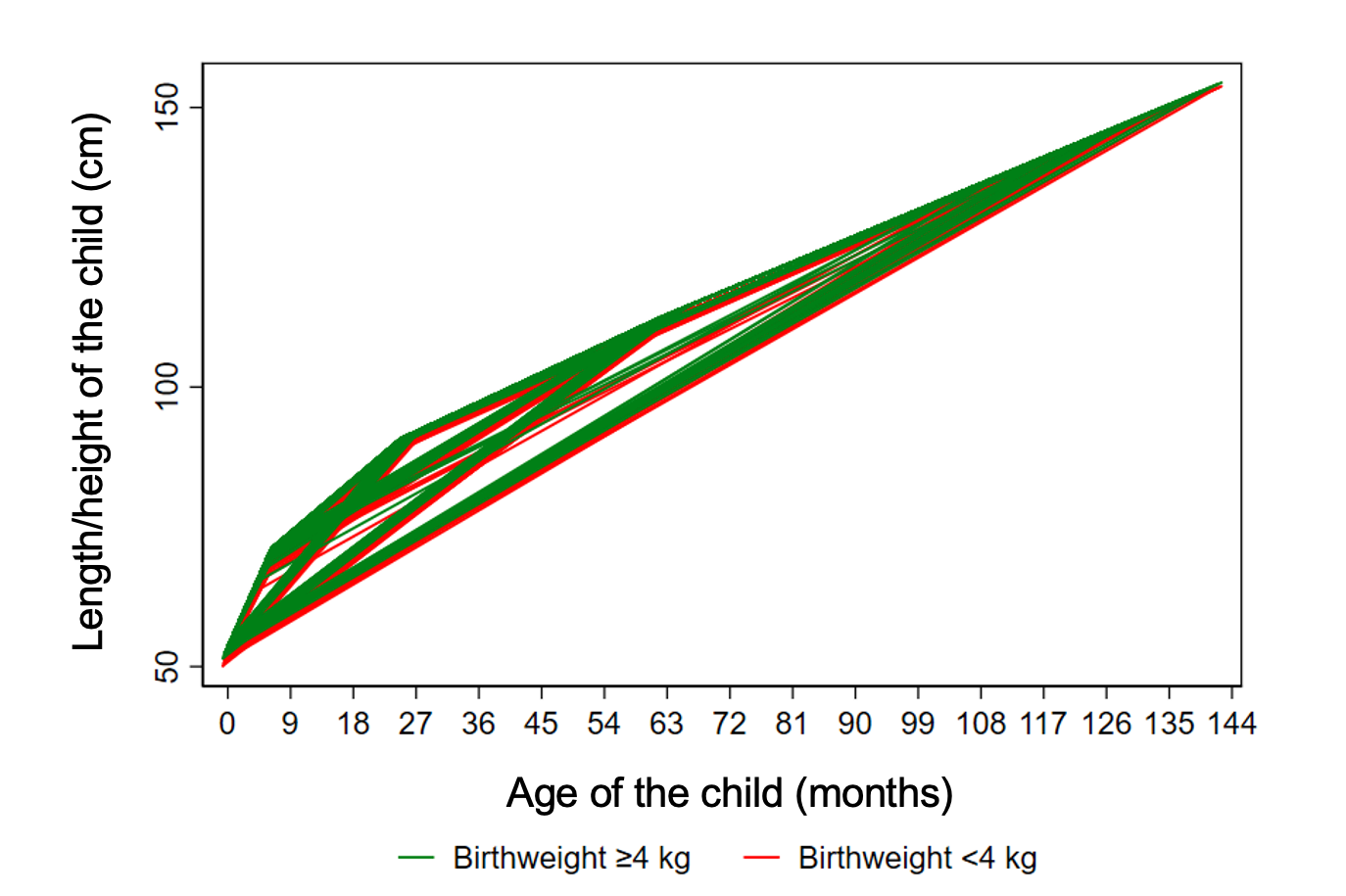
**

**Supplementary Figure 2:** Length/height trajectories from birth to 10 years of age for those born with and without macrosomia (birthweight ≥4 kg).

**
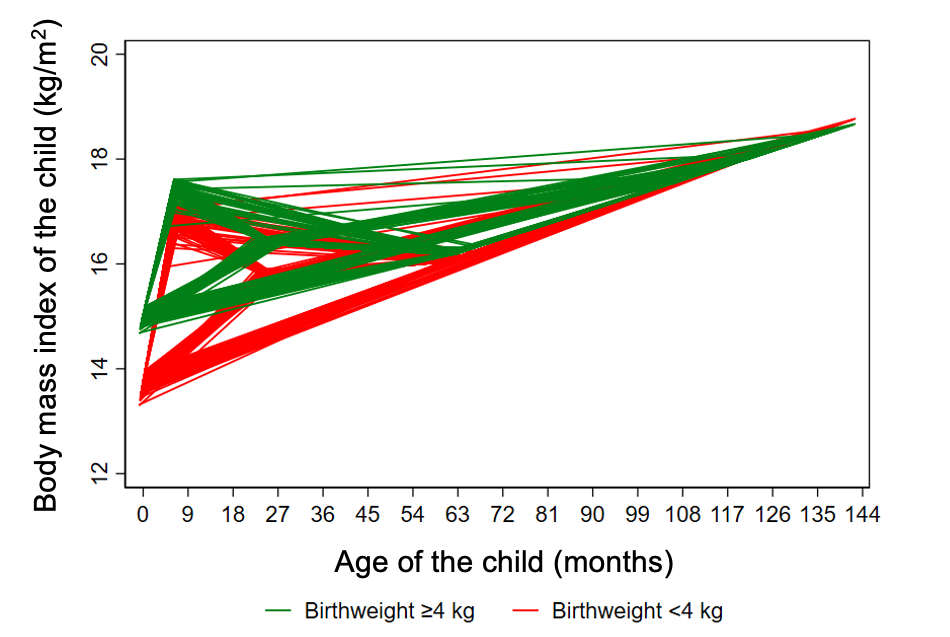
**

**Supplementary Figure 3:** BMI trajectories from birth to 10 years of age for those born with and without macrosomia (birthweight ≥4 kg).

**
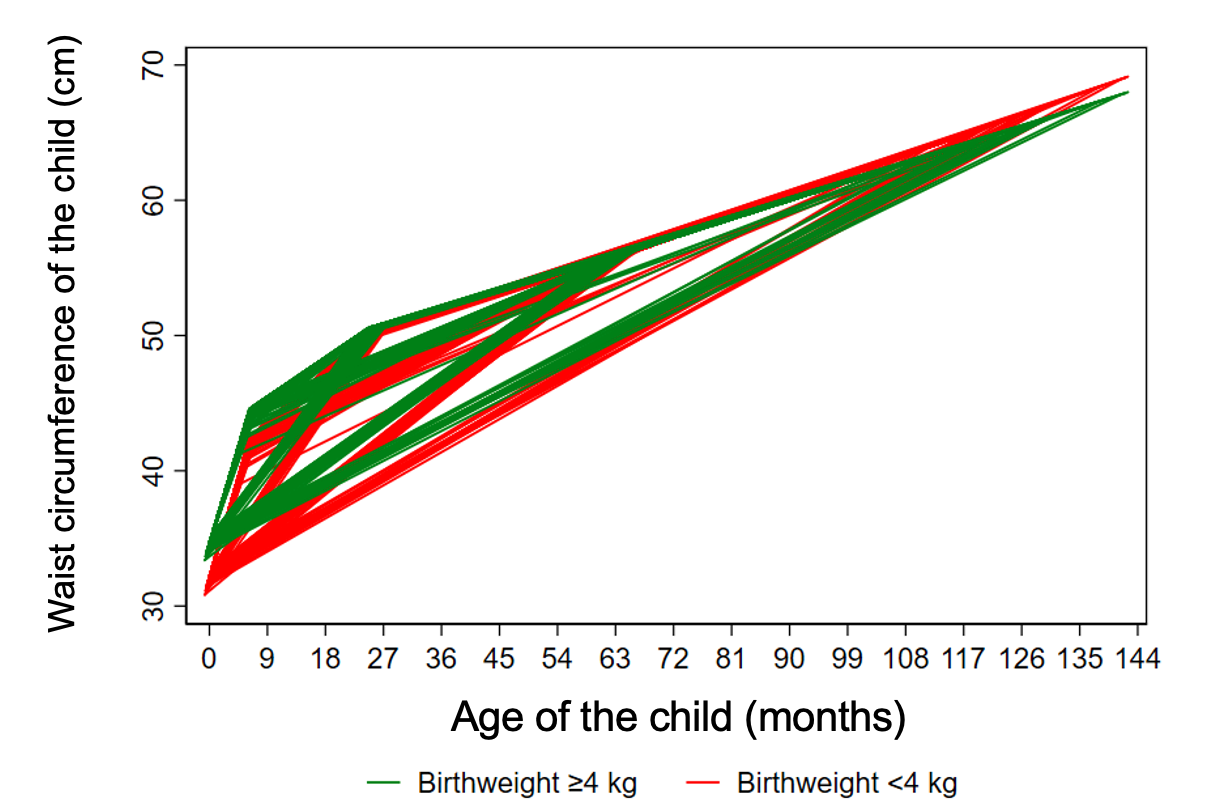
**

**Supplementary Figure 4:** Waist circumference trajectories from birth to 10 years of age for those born with and without macrosomia (birthweight ≥4 kg).

**Supplementary Tables**

| Supplementary Table 1: Differences in maternal and child characteristics between those born ≥4.5 kg compared to <4.5 kg. | | | | | |
| --- | --- | --- | --- | --- | --- |
|  | **Birthweight ≥4.5 kg** | | **Birthweight <4.5 kg** | | ***P*** |
|  | **N** | **Mean (SD) / Median (IQR) / n (%)** | **N** | **Mean (SD) / Median (IQR) / n (%)** |  |
| Age at delivery (years) | 52 | 32.8 (29.9, 34.9) | 285 | 33.2 (30.8, 35.3) | 0.58 |
| HP index | 52 | 7.1 (1.1, 9.77) | 285 | 7.0 (-1.25, 11.9) | 0.98 |
| Early pregnancy BMI (kg/m^2^) | 52 | 27.3 (23.7, 32.7) | 285 | 25.2 (22.9, 27.7) | 0.002 |
| Paternal BMI (kg/m^2^) | 52 | 28.1 (25.3, 29.4) | 285 | 27.2 (25.0, 29.4) | 0.32 |
| *Ethnicity* |  |  |  |  |  |
| White Irish, n (%) | 52 | 51 (98.1) | 285 | 256 (89.8) | 0.06 |
| Other, n (%) | 52 | 1 (1.9) | 285 | 29 (10.2) |  |
| *Gestational weight gain* |  |  |  |  |  |
| Total (kg) | 52 | 14.3 (5.27) | 285 | 13.2 (4.2) | 0.13 |
| Inadequate, n (%) | 52 | 3 (5.8) | 285 | 45 (15.8) | 0.009 |
| Adequate, n (%) | 52 | 15 (28.8) | 285 | 117 (41.1) |  |
| Excessive, n (%) | 52 | 34 (65.4) | 285 | 123 (43.2) |  |
| *Metabolic complication in pregnancy* | | | | | |
| Yes, n (%) | 52 | 15 (28.8) | 285 | 79 (27.7) | 0.86 |
| No, n (%) | 52 | 37 (71.2) | 285 | 206 (72.3) |  |
| *Study group allocation* |  |  |  |  |  |
| Intervention, n (%) | 52 | 32 (61.5) | 285 | 143 (50.2) | 0.13 |
| Control, n (%) | 52 | 20 (38.5) | 285 | 142 (49.8) |  |
| *Smoking in pregnancy* |  |  |  |  |  |
| Yes, n (%) | 52 | 1 (1.9) | 285 | 13 (4.6) | 0.38 |
| No, n (%) | 52 | 51 (98.1) | 285 | 272 (95.4) |  |
| *Adherence to a special diet in pregnancy* | | | | | |
| Yes, n (%) | 52 | 14 (26.9) | 285 | 43 (15.1) | 0.036 |
| No, n (%) | 52 | 38 (73.1) | 285 | 242 (84.9) |  |
| Physical activity level (METs/day) | 52 | 325.0 (198.0, 504.0) | 285 | 344.0 (198.0, 560.0) | 0.67 |
| *Child sex* |  |  |  |  |  |
| Male, n (%) | 52 | 34 (65.4) | 285 | 124 (43.5) | 0.004 |
| Female, n (%) | 52 | 18 (34.6) | 285 | 161 (56.5) |  |
| *Exposure to breast milk* |  |  |  |  |  |
| Never, n (%) | 24 | 11 (45.8) | 159 | 59 (37.1) | 0.43 |
| <2 months, n (%) | 24 | 1 (4.2) | 159 | 17 (10.7) |  |
| ≥2 and <4 months, n (%) | 24 | 5 (20.8) | 159 | 21 (13.2) |  |
| ≥4 months, n (%) | 24 | 7 (29.2) | 159 | 59 (20.7) |  |
| Birthweight (kg) | 52 | 4.68 (4.58, 4.84) | 285 | 3.96 (3.72, 4.16) | <0.001 |
| Birthweight centile | 52 | 97.4 (94.1, 99.4) | 285 | 77.4 (57.2, 89.1) | <0.001 |
| GA at delivery (days) | 52 | 285.5 (282.0, 291.7) | 285 | 282.0 (277.0, 827.0) | <0.001 |
| Abbreviations: SD Standard deviation; IQR Interquartile range; HP Haase and Pratschke Deprivation index; BMI Body mass index; GA Gestational age. Results presented as mean (SD) for normally distributed variables, median (IQR 25^th^, 75^th^ percentile) for non-normally distributed variables, or frequency and percentage n (%) for categorical variables. P-values determined using independent t-tests for normally distributed variables, Mann Whitney-U tests for non-normally distributed variables, or Chi-square tests for categorical variables; P<0.05 considered statistically significant. N = total population; n = frequency. | | | | | |

| Supplementary Table 2: Differences in maternal and child characteristics between those born ≥90^th^ centile compared to <90^th^ centile. | | | | | |
| --- | --- | --- | --- | --- | --- |
|  | **Birthweight ≥90^th^ centile** | | **Birthweight <90^th^ centile** | | ***P*** |
|  | **N** | **Mean (SD) / Median (IQR) / n (%)** | **N** | **Mean (SD) / Median (IQR) / n (%)** |  |
| Age at delivery (years) | 115 | 32.6 (29.9, 34.9) | 222 | 33.6 (31.2, 35.4) | 0.10 |
| HP index | 115 | 6.7 (-0.4, 10.4) | 222 | 7.25 (-1.4, 12.2) | 0.54 |
| Early pregnancy BMI (kg/m^2^) | 115 | 24.7 (23.2, 29.0) | 222 | 25.5 (23.1, 28.2) | 0.80 |
| Paternal BMI (kg/m^2^) | 115 | 27.7 (25.4, 29.6) | 222 | 27.0 (24.9, 29.2) | 0.09 |
| *Ethnicity* |  |  |  |  |  |
| White Irish, n (%) | 115 | 105 (91.3) | 222 | 202 (91.0) | 0.92 |
| Other, n (%) | 115 | 10 (8.7) | 222 | 20 (9.0) |  |
| *Gestational weight gain* |  |  |  |  |  |
| Total (kg) | 115 | 14.1 (4.2) | 222 | 13.1 (4.46) | 0.038 |
| Inadequate, n (%) | 115 | 8 (7.0) | 222 | 40 (18.0) | 0.020 |
| Adequate, n (%) | 115 | 47 (40.9) | 222 | 85 (38.3) |  |
| Excessive, n (%) | 115 | 60 (52.2) | 222 | 97 (43.7) |  |
| *Metabolic complication in pregnancy* | | | | | |
| Yes, n (%) | 115 | 34 (29.6) | 222 | 60 (27.0) | 0.62 |
| No, n (%) | 115 | 81 (70.4) | 222 | 162 (73.0) |  |
| *Study group allocation* |  |  |  |  |  |
| Intervention, n (%) | 115 | 69 (60.0) | 222 | 106 (47.7) | 0.033 |
| Control, n (%) | 115 | 46 (40.0) | 222 | 116 (52.3) |  |
| *Smoking in pregnancy* |  |  |  |  |  |
| Yes, n (%) | 115 | 3 (2.6) | 222 | 11 (5.0) | 0.30 |
| No, n (%) | 115 | 112 (97.4) | 222 | 211 (95.0) |  |
| *Adherence to a special diet in pregnancy* | | | | | |
| Yes, n (%) | 115 | 21 (18.3) | 222 | 36 (16.2) | 0.63 |
| No, n (%) | 115 | 94 (81.7) | 222 | 186 (83.8) |  |
| Physical activity level (METs/day) | 115 | 320.0 (198.0, 560.0) | 222 | 351.0 (208.5, 543.0) | 0.36 |
| *Child sex* |  |  |  |  |  |
| Male, n (%) | 115 | 57 (49.6) | 222 | 101 (45.5) | 0.47 |
| Female, n (%) | 115 | 58 (50.4) | 222 | 121 (54.5) |  |
| *Exposure to breast milk* |  |  |  |  |  |
| Never, n (%) | 59 | 27 (45.8) | 124 | 43 (34.7) | 0.47 |
| <2 months, n (%) | 59 | 6 (10.2) | 124 | 12 (9.7) |  |
| ≥2 and <4 months, n (%) | 59 | 8 (13.6) | 124 | 18 (14.5) |  |
| ≥4 months, n (%) | 59 | 18 (30.5) | 124 | 51 (41.1) |  |
| Birthweight (kg) | 115 | 4.42 (0.38) | 222 | 3.87 (0.32) | <0.001 |
| Birthweight centile | 115 | 95.8 (93.6, 98.5) | 222 | 70.6 (48.7, 80.2) | <0.001 |
| GA at delivery (days) | 115 | 281.0 (277.0, 286.0) | 222 | 284.0 (278.0, 289.0) | 0.004 |
| Abbreviations: SD Standard deviation; IQR Interquartile range; HP Haase and Pratschke Deprivation index; BMI Body mass index; GA Gestational age. Results presented as mean (SD) for normally distributed variables, median (IQR 25^th^, 75^th^ percentile) for non-normally distributed variables, or frequency and percentage n (%) for categorical variables. P-values determined using independent t-tests for normally distributed variables, Mann Whitney-U tests for non-normally distributed variables, or Chi-square tests for categorical variables; P<0.05 considered statistically significant. N = total population; n = frequency. | | | | | |

| Supplementary Table 3: Anthropometric characteristics from birth to 10 years of age. | | | |
| --- | --- | --- | --- |
|  | **N** | **Mean (SD)** | **Median**  **(IQR 25^th^, 75^th^ percentile)** |
| Weight (kg) |  |  |  |
| Birth | 337 | 4.05 (0.43) | 4.02 (3.77, 4.34) |
| 6 months | 129 | 8.33 (1.33) | 8.19 (7.64, 8.70) |
| 2 years | 160 | 13.07 (1.57) | 12.9 (11.8, 14.0) |
| 5 years | 180 | 20.1 (2.37) | 19.9 (18.4, 22.0) |
| 10 years | 203 | 36.6 (8.60) | 34.6 (30.8, 41.2) |
| Length/height (cm) |  |  |  |
| Birth | 316 | 53.01 (2.06) | 53.0 (51.5, 54.0) |
| 6 months | 129 | 69.6 (2.85) | 69.5 (67.5, 71.0) |
| 2 years | 160 | 90.0 (3.43) | 90.0 (87.5, 92.0) |
| 5 years | 179 | 111.7 (4.30) | 111.5 (108.8, 114.7) |
| 10 years | 203 | 142.0 (7.23) | 141.8 (136.7, 146.9) |
| BMI (kg/m^2^) |  |  |  |
| Birth | 316 | 14.4 (1.34) | 14.3 (13.5, 15.1) |
| 6 months | 129 | 17.1 (2.62) | 16.8 (15.7, 17.9) |
| 2 years | 159 | 16.1 (1.55) | 15.9 (15.1, 16.9) |
| 5 years | 179 | 16.1 (1.26) | 16.1 (15.2, 16.9) |
| 10 years | 203 | 17.9 (2.94) | 17.1 (15.8, 19.6) |
| Waist circumference (cm) | | | |
| Birth | 91 | 33.5 (2.40) | 33.5 (32.0, 35.2) |
| 6 months | 129 | 43.2 (3.53) | 43.4 (41.2, 45.0) |
| 2 years | 159 | 50.1 (3.62) | 50.0 (47.5, 52.4) |
| 5 years | 178 | 55.6 (3.66) | 55.5 (53.0, 58.0) |
| 10 years | 203 | 64.9 (9.19) | 62.5 (58.8, 69.2) |
| Abbreviations: SD Standard deviation; IQR Interquartile range; BMI Body mass index. All data presented as mean (SD) and median (IQR 25^th^, 75^th^ percentile). | | | |

| Supplementary Table 4: Crude and adjusted mean trajectory and mean difference in trajectory of weight from 6 months to 10 years of age for birthweight ≥4 kg, estimated from multilevel linear spline models. | | |
| --- | --- | --- |
|  | **Mean weight**  **trajectory (95% CI) (kg) for those born <4 kg** | **Difference (95% CI) in mean weight trajectory**  **for those born ≥4 kg** |
| *Unadjusted* |  |  |
| Weight at 6 months (kg) | 8.0 (7.70, 8.29) | 0.69 (0.28, 1.10) |
| Δ 6 months – 2 years (kg/week) | 0.05 (0.05, 0.06) | 0.0008 (-0.005, 0.007) |
| Weight at 2 years (kg) | 12.5 (12.1, 12.8) | 0.76 (0.27, 1.24) |
| Δ 2 years – 5 years (kg/week) | 0.04 (0.03, 0.04) | -0.0009 (-0.005, 0.003) |
| Weight at 5 years (kg) | 19.1 (18.6, 19.6) | 0.61 (-0.03, 1.26) |
| Δ 5 years – 10 years (kg/week) | 0.06 (0.06, 0.07) | -0.002 (-0.01, 0.006) |
| Weight at 10 years (kg) | 37.1 (35.5, 38.6) | 0.09 (-2.03, 2.21) |
| *Adjusted* |  |  |
| Weight at 6 months (kg) | 7.39 (6.42, 8.37) | 0.50 (0.08, 0.93) |
| Δ 6 months – 2 years (kg/week) | 0.06 (0.04, 0.07) | 0.001 (-0.005, 0.008) |
| Weight at 2 years (kg) | 12.3 (11.3, 13.4) | 0.64 (0.14, 1.14) |
| Δ 2 years – 5 years (kg/week) | 0.04 (0.03, 0.05) | -0.001 (-0.006, 0.002) |
| Weight at 5 years (kg) | 19.04 (17.7, 20.3) | 0.37 (-0.28, 1.03) |
| Δ 5 years – 10 years (kg/week) | 0.07 (0.05, 0.08) | -0.001 (-0.009, 0.006) |
| Weight at 10 years (kg) | 38.3 (34.3, 42.3) | -0.05 (-2.11, 1.99) |
| Abbreviations: CI Confidence interval; Δ the change. Adjusted models controlled for original study group, child sex, HP index, maternal age at delivery, maternal ethnicity, gestational weight gain, maternal smoking in pregnancy, maternal physical activity in pregnancy, adherence to a special diet in pregnancy, metabolic complications in pregnancy, and paternal BMI. Mean trajectory is centred on the mean of the first measurement (6 months) as the reference category. N = 259 | | |

| Supplementary Table 5: Crude and adjusted mean trajectory and mean difference in trajectory of WC from birth to 10 years of age for birthweight (≥4.5 kg; ≥90^th^ centile; continuous), estimated from multilevel linear spline models. | | | | | | |
| --- | --- | --- | --- | --- | --- | --- |
|  | **Mean WC**  **trajectory (95% CI) (cm) for those born <4.5 kg** | **Difference (95% CI) in mean WC trajectory for those born ≥4.5 kg** | **Mean WC trajectory (95% CI) (cm) for those born <90^th^ birthweight centile** | **Difference (95% CI) in mean WC trajectory for those born ≥90^th^ birthweight centile** | **Mean WC**  **trajectory (95% CI) (cm)** | **Difference (95% CI) in mean WC trajectory per 1-unit higher birthweight (kg)** |
| *Unadjusted* |  |  |  |  |  |  |
| WC at birth (cm) | 33.2 (32.6, 33.7) | 2.12 (0.66, 3.58) | 32.6 (32.0, 33.3) | 2.49 (1.41, 3.57) | 33.4 (32.9, 33.9) | 2.86 (1.82, 3.89) |
| Δ Birth to 6 months (cm/week) | 0.37 (0.34, 0.40) | -0.01 (-0.09, 0.07) | 0.38 (0.34, 0.42) | -0.02 (-0.08, 0.03) | 0.37 (0.34, 0.40) | -0.005 (-0.06, 0.05) |
| WC at 6 months (cm) | 42.9 (42.3, 43.5) | 1.81 (0.13, 3.49) | 42.6 (42.0, 43.3) | 1.81 (0.55, 3.08) | 43.2 (42.6, 43.7) | 2.72 (1.44, 4.00) |
| Δ 6 months – 2 years (cm/week) | 0.08 (0.07, 0.09) | -0.01 (-0.03, 0.01) | 0.09 (0.08, 0.10) | -0.02 (-0.04, -0.002) | 0.08 (0.07, 0.09) | -0.03 (-0.05, -0.009) |
| WC at 2 years (cm) | 49.7 (49.1, 50.3) | 1.00 (-0.62, 2.63) | 49.8 (49.1, 50.5) | 0.09 (-1.06, 1.24) | 49.9 (49.3, 50.4) | 0.38 (-0.87, 1.64) |
| Δ 2 years – 5 years (cm/week) | 0.03 (0.02, 0.03) | 0.005 (-0.007, 0.01) | 0.03 (0.02, 0.03) | 0.002 (-0.006, 0.01) | 0.03 (0.02, 0.03) | 0.003 (-0.007, 0.01) |
| WC at 5 years (cm) | 54.6 (54.0, 55.2) | 1.89 (0.30, 3.49) | 54.7 (54.0, 55.3) | 0.51 (-0.67, 1.70) | 54.8 (54.3, 55.4) | 0.88 (-0.46, 2.23) |
| Δ 5 years – 10 years (cm/week) | 0.03 (0.03, 0.04) | -0.0008 (-0.01, 0.01) | 0.03 (0.03, 0.04) | -0.004 (-0.01, 0.006) | 0.03 (0.03, 0.04) | -0.003 (-0.01, 0.008) |
| WC at 10 years (cm) | 64.3 (62.9, 65.6) | 1.67 (-1.93, 5.28) | 64.7 (63.2, 66.2) | -0.58 (-3.24, 2.08) | 64.5 (63.2, 65.7) | 0.04 (-2.97, 3.06) |
| *Adjusted* |  |  |  |  |  |  |
| WC at birth (cm) | 33.1 (31.2, 35.1) | 2.09 (0.61, 3.58) | 32.1 (30.1, 34.0) | 2.41 (1.29, 3.53) | 34.2 (32.3, 36.1) | 2.99 (1.90, 4.08) |
| Δ Birth to 6 months (cm/week) | 0.35 (0.22, 0.48) | -0.04 (-0.13, 0.04) | 0.36 (0.23, 0.49) | -0.03 (-0.10, 0.02) | 0.33 (0.20, 0.45) | -0.02 (-0.09, 0.04) |
| WC at 6 months (cm) | 42.4 (39.6, 45.1) | 1.04 (-0.72, 2.80) | 41.6 (38.8, 44.3) | 1.38 (0.06, 2.70) | 42.8 (40.1, 45.4) | 2.39 (1.00, 3.78) |
| Δ 6 months – 2 years (cm/week) | 0.09 (0.05, 0.13) | 0.003 (-0.02, 0.03) | 0.10 (0.05, 0.14) | -0.01 (-0.03, 0.004) | 0.09 (0.04, 0.13) | -0.02 (-0.04, -0.001) |
| WC at 2 years (cm) | 49.8 (47.3, 52.3) | 1.30 (-0.39, 3.00) | 49.7 (47.2, 52.3) | 0.14 (-1.05, 1.34) | 50.0 (47.5, 52.4) | 0.57 (-0.74, 1.89) |
| Δ 2 years – 5 years (cm/week) | 0.03 (0.01, 0.05) | 0.003 (-0.02, 0.03) | 0.03 (0.01, 0.05) | 0.0008 (-0.008, 0.01) | 0.03 (0.01, 0.05) | 0.001 (-0.008, 0.01) |
| WC at 5 years (cm) | 54.9 (52.7, 57.0) | 1.89 (0.28, 3.50) | 54.7 (52.6, 56.9) | 0.27 (-0.92, 1.46) | 55.1 (52.9, 57.3) | 0.87 (-0.51, 2.27) |
| Δ 5 years – 10 years (cm/week) | 0.03 (0.01, 0.05) | -0.003 (-0.01, 0.01) | 0.03 (0.02, 0.05) | -0.005 (-0.01, 0.004) | 0.03 (0.01, 0.05) | -0.004 (-0.01, 0.007) |
| WC at 10 years (cm) | 64.4 (59.8, 69.0) | 0.94 (-2.60, 4.49) | 64.7 (60.1, 69.3) | -1.19 (-3.73, 1.34) | 64.3 (59.7, 69.0) | -0.28 (-3.25, 2.69) |
| Abbreviations: WC Waist circumference; CI Confidence interval; Δ the change. Adjusted models controlled for original study group, child sex, HP index, maternal age at delivery, maternal ethnicity, gestational weight gain, maternal smoking in pregnancy, maternal physical activity in pregnancy, adherence to a special diet in pregnancy, metabolic complications in pregnancy, and paternal BMI. Mean trajectory is centred on the mean of the first measurement (birth) as the reference category. N = 271 | | | | | | |

| Supplementary Table 6: Crude and adjusted mean trajectory and mean difference in trajectory of weight from birth to 10 years of age for birthweight (≥4.5 kg; ≥90^th^ centile; continuous), estimated from multilevel linear spline models. | | | | | | |
| --- | --- | --- | --- | --- | --- | --- |
|  | **Mean weight**  **trajectory (95% CI) (kg) for those born <4.5 kg** | **Difference (95% CI) in mean weight trajectory**  **for those born ≥4.5 kg** | **Mean weight**  **trajectory (95% CI) (kg) for those born <90^th^ birthweight centile** | **Difference (95% CI) in mean weight trajectory**  **for those born ≥90^th^ birthweight centile** | **Mean weight**  **trajectory (95% CI) (kg)** | **Difference (95% CI) in mean weight trajectory**  **per 1-unit higher birthweight (kg)** |
| *Unadjusted* |  |  |  |  |  |  |
| Weight at birth (kg) | 3.94 (3.91, 3.98) | 0.73 (0.64, 0.82) | 3.84 (3.81, 3.88) | 0.61 (0.55, 0.68) | 4.05 (4.04, 4.07) | 0.86 (0.82, 0.90) |
| Δ Birth to 6 months (kg/week) | 0.16 (0.15, 0.17) | -0.005 (-0.02, 0.01) | 0.16 (0.15, 0.17) | 0.01 (-0.002, 0.02) | 0.14 (0.14, 0.15) | 0.01 (-0.003, 0.03) |
| Weight at 6 months (kg) | 8.24 (8.03, 8.45) | 0.59 (0.02, 1.15) | 8.11 (7.88, 8.34) | 0.97 (0.55, 1.40) | 7.92 (7.73, 8.12) | 1.22 (0.77, 1.67) |
| Δ 6 months – 2 years (kg/week) | 0.05 (0.05, 0.06) | 0.004 (-0.005, 0.01) | 0.05 (0.05, 0.06) | -0.005 (-0.01, 0.002) | 0.06 (0.05, 0.06) | -0.004 (-0.01, 0.003) |
| Weight at 2 years (kg) | 12.7 (12.4, 13.0) | 0.90 (0.11, 1.69) | 12.6 (12.3, 12.9) | 0.57 (0.006, 1.15) | 12.7 (12.4, 13.1) | 0.84 (0.18, 1.51) |
| Δ 2 years – 5 years (kg/week) | 0.04 (0.04, 0.05) | 0.001 (-0.003, 0.006) | 0.04 (0.04, 0.05) | -0.0005 (-0.003, 0.002) | 0.04 (0.04, 0.05) | 0.0006 (-0.003, 0.004) |
| Weight at 5 years (kg) | 19.5 (19.1, 19.8) | 1.13 (0.21, 2.04) | 19.5 (19.1, 19.9) | 0.49 (-0.18, 1.16) | 19.6 (19.2, 19.9) | 0.95 (0.16, 1.73) |
| Δ 5 years – 10 years (kg/week) | 0.06 (0.06, 0.07) | 0.006 (-0.003, 0.01) | 0.06 (0.06, 0.07) | 0.001 (-0.006, 0.008) | 0.06 (0.06, 0.07) | 0.001 (-0.007, 0.01) |
| Weight at 10 years (kg) | 36.2 (35.2, 37.2) | 2.91 (0.13, 5.69) | 36.4 (35.2, 37.5) | 0.81 (-1.25, 2.88) | 36.6 (35.6, 37.6) | 1.30 (-1.05, 3.66) |
| *Adjusted* |  |  |  |  |  |  |
| Weight at birth (kg) | 3.90 (3.78, 4.03) | 0.70 (0.60, 0.79) | 3.68 (3.57, 3.79) | 0.60 (0.53, 0.66) | 4.07 (4.01, 4.13) | 0.86 (0.82, 0.90) |
| Δ Birth to 6 months (kg/week) | 0.14 (0.10, 0.17) | -0.01 (-0.03, 0.006) | 0.14 (0.10, 0.17) | 0.01 (-0.004, 0.02) | 0.11 (0.08, 0.14) | 0.007 (-0.01, 0.02) |
| Weight at 6 months (kg) | 7.60 (6.69, 8.50) | 0.28 (-0.30, 0.87) | 7.40 (6.50, 8.31) | 0.92 (0.48, 1.36) | 7.04 (6.16, 7.92) | 1.06 (0.57, 1.54) |
| Δ 6 months – 2 years (kg/week) | 0.06 (0.04, 0.08) | 0.007 (-0.002, 0.01) | 0.06 (0.04, 0.08) | -0.003 (-0.01, 0.003) | 0.07 (0.05, 0.09) | -0.002 (-0.01, 0.006) |
| Weight at 2 years (kg) | 12.6 (11.4, 13.8) | 0.90 (0.08, 1.72) | 12.3 (11.1, 13.5) | 0.63 (0.04, 1.22) | 12.9 (11.7, 14.2) | 0.88 (0.18, 1.58) |
| Δ 2 years – 5 years (kg/week) | 0.04 (0.03, 0.05) | 0.001 (-0.003, 0.005) | 0.04 (0.03, 0.05) | -0.001 (-0.005, 0.001) | 0.04 (0.03, 0.05) | -0.00003 (-0.003, 0.004) |
| Weight at 5 years (kg) | 19.7 (18.4, 20.9) | 1.08 (0.16, 2.00) | 19.5 (18.2, 20.7) | 0.34 (-0.32, 1.02) | 19.9 (18.6, 21.2) | 0.88 (0.06, 1.69) |
| Δ 5 years – 10 years (kg/week) | 0.07 (0.05, 0.08) | 0.005 (-0.004, 0.01) | 0.07 (0.05, 0.08) | -0.0006 (-0.007, 0.006) | 0.07 (0.05, 0.08) | 0.0009 (-0.007, 0.009) |
| Weight at 10 years (kg) | 37.9 (34.4, 41.5) | 2.45 (-0.29, 5.19) | 37.8 (34.2, 41.4) | 0.18 (-1.79, 2.16) | 38.1 (34.5, 41.7) | 1.12 (-1.21, 3.45) |
| Abbreviations: CI Confidence interval; Δ the change. Adjusted models controlled for original study group, child sex, HP index, maternal age at delivery, maternal ethnicity, gestational weight gain, maternal smoking in pregnancy, maternal physical activity in pregnancy, adherence to a special diet in pregnancy, metabolic complications in pregnancy, and paternal BMI. Mean trajectory is centred on the mean of the first measurement (birth) as the reference category. N = 337 | | | | | | |

| Supplementary Table 7: Crude and adjusted mean trajectory and mean difference in trajectory of length/height from birth to 10 years of age for birthweight (≥4.5 kg; ≥90^th^ centile; continuous), estimated from multilevel linear spline models. | | | | | | |
| --- | --- | --- | --- | --- | --- | --- |
|  | **Mean length/height**  **trajectory (95% CI) (cm) for those born <4.5 kg** | **Difference (95% CI) in mean length/height trajectory for those born ≥4.5 kg** | **Mean length/height**  **trajectory (95% CI) (cm) for those born <90^th^ birthweight centile** | **Difference (95% CI) in mean length/height trajectory for those born ≥90^th^ birthweight centile** | **Mean length/height**  **trajectory (95% CI) (cm)** | **Difference (95% CI) in mean length/height trajectory per 1-unit higher birthweight (kg)** |
| *Unadjusted* |  |  |  |  |  |  |
| Length at birth (cm) | 52.7 (52.5, 53.01) | 1.46 (0.89, 2.03) | 52.5 (52.2, 52.7) | 1.50 (1.08, 1.91) | 53.0 (52.8, 53.2) | 1.80 (1.33, 2.26) |
| Δ Birth to 6 months (cm/week) | 0.63 (0.62, 0.65) | 0.03 (-0.01, 0.08) | 0.64 (0.62, 0.66) | 0.002 (-0.03, 0.03) | 0.64 (0.62, 0.66) | 0.01 (-0.02, 0.05) |
| Length at 6 months (cm) | 69.4 (68.9, 69.9) | 2.32 (1.07, 3.58) | 69.2 (68.7, 69.8) | 1.57 (0.61, 2.52) | 69.7 (69.3, 70.1) | 2.27 (1.28, 3.26) |
| Δ 6 months – 2 years (cm/week) | 0.24 (0.24, 0.25) | -0.001 (-0.02, 0.01) | 0.24 (0.23, 0.25) | 0.003 (-0.01, 0.01) | 0.24 (0.24, 0.25) | -0.003 (-0.01, 0.01) |
| Height at 2 years (cm) | 88.8 (88.2, 89.3) | 2.17 (0.75, 3.59) | 88.5 (87.9, 89.1) | 1.84 (0.80, 2.87) | 89.1 (88.6, 89.6) | 1.96 (0.82, 3.10) |
| Δ 2 years – 5 years (cm/week) | 0.13 (0.13, 0.14) | -0.0004 (-0.01, 0.009) | 0.13 (0.12, 0.14) | -0.003 (-0.01, 0.003) | 0.13 (0.13, 0.14) | -0.002 (-0.01, 0.005) |
| Height at 5 years (cm) | 109.8 (109.2, 110.4) | 2.10 (0.50, 3.69) | 109.7 (109.0, 110.4) | 1.28 (0.09, 2.48) | 110.1 (109.5, 110.7) | 1.61 (0.27, 2.95) |
| Δ 5 years – 10 years (cm/week) | 0.12 (0.11, 0.12) | 0.004 (-0.001, 0.01) | 0.12 (0.11, 0.12) | 0.001 (-0.002, 0.005) | 0.12 (0.11, 0.12) | -0.0005 (-0.005, 0.004) |
| Height at 10 years (cm) | 141.1 (140.3, 141.8) | 3.24 (1.28, 5.20) | 141.0 (140.1, 141.8) | 1.68 (0.21, 3.14) | 141.5 (140.8, 142.2) | 1.46 (-0.18, 3.10) |
| *Adjusted* |  |  |  |  |  |  |
| Length at birth (cm) | 52.9 (52.1, 53.6) | 1.28 (0.72, 1.85) | 52.3 (51.6, 53.1) | 1.43 (1.02, 1.83) | 53.2 (52.5, 54.0) | 1.68 (1.20, 2.16) |
| Δ Birth to 6 months (cm/week) | 0.57 (0.49, 0.64) | 0.01 (-0.04, 0.06) | 0.57 (0.49, 0.65) | -0.004 (-0.04, 0.03) | 0.56 (0.49, 0.64) | -0.01 (-0.05, 0.03) |
| Length at 6 months (cm) | 67.8 (65.8, 69.7) | 1.56 (0.27, 2.85) | 67.3 (65.2, 69.3) | 1.32 (0.34, 2.29) | 68.0 (66.1, 70.0) | 1.40 (0.35, 2.46) |
| Δ 6 months – 2 years (cm/week) | 0.25 (0.22, 0.28) | 0.004 (-0.01, 0.02) | 0.24 (0.21, 0.28) | 0.004 (-0.01, 0.01) | 0.25 (0.22, 0.28) | 0.001 (-0.01, 0.01) |
| Height at 2 years (cm) | 87.5 (85.5, 89.6) | 1.88 (0.46, 3.31) | 86.7 (84.5, 88.8) | 1.64 (0.61, 2.66) | 87.8 (85.7, 89.9) | 1.51 (0.36, 2.66) |
| Δ 2 years – 5 years (cm/week) | 0.14 (0.13, 0.16) | -0.00004 (-0.01, 0.01) | 0.14 (0.13, 0.16) | -0.003 (-0.01, 0.004) | 0.14 (0.12, 0.16) | -0.001 (-0.009, 0.007) |
| Height at 5 years (cm) | 110.2 (108.1, 112.3) | 1.88 (0.27, 3.48) | 109.7 (107.6, 111.9) | 1.15 (-0.02, 2.33) | 110.4 (108.2, 112.6) | 1.29 (-0.08, 2.67) |
| Δ 5 years – 10 years (cm/week) | 0.12 (0.11, 0.13) | 0.005 (-0.0008, 0.01) | 0.12 (0.11, 0.13) | 0.001 (-0.003, 0.005) | 0.12 (0.11, 0.13) | 0.0004 (-0.004, 0.005) |
| Height at 10 years (cm) | 142.4 (139.8, 145.0) | 3.24 (1.27, 5.21) | 141.8 (139.2, 144.4) | 1.46 (0.03, 2.89) | 142.6 (140.0, 145.2) | 1.40 (-0.26, 3.07) |
| Abbreviations: CI Confidence interval; Δ the change. Adjusted models controlled for original study group, child sex, HP index, maternal age at delivery, maternal ethnicity, gestational weight gain, maternal smoking in pregnancy, maternal physical activity in pregnancy, adherence to a special diet in pregnancy, metabolic complications in pregnancy, and paternal BMI. Mean trajectory is centred on the mean of the first measurement (birth) as the reference category. N = 331 | | | | | | |

| Supplementary Table 8: Crude and adjusted mean trajectory and mean difference in trajectory of BMI from birth to 10 years of age for birthweight (≥4.5 kg; ≥90^th^ centile; continuous), estimated from multilevel linear spline models. | | | | | | |
| --- | --- | --- | --- | --- | --- | --- |
|  | **Mean BMI**  **trajectory (95% CI) (kg/m^2^) for those born <4.5 kg** | **Difference (95% CI) in mean BMI trajectory for those born ≥4.5 kg** | **Mean BMI trajectory (95% CI) (kg/m^2^) for those born <90^th^ birthweight centile** | **Difference (95% CI) in mean BMI trajectory for those born ≥90^th^ birthweight centile** | **Mean BMI trajectory (95% CI) (kg/m^2^) for those born <90^th^ birthweight centile** | **Difference (95% CI) in mean BMI trajectory for those born ≥90^th^ birthweight centile** |
| *Unadjusted* |  |  |  |  |  |  |
| BMI at birth (kg/m^2^) | 14.1 (14.0, 14.3) | 1.67 (1.26, 2.08) | 14.0 (13.8, 14.1) | 1.2 (0.89, 1.5) | 14.4 (14.3, 14.5) | 2.07 (1.77, 2.38) |
| Δ Birth – 6 months (kg/m^2^/week) | 0.11 (0.10, 0.13) | -0.06 (-0.09, -0.02) | 0.11 (0.09, 0.12) | -0.004 (-0.03, 0.02) | 0.1 (0.09, 0.11) | -0.04 (-0.06, -0.01) |
| BMI at 6 months (kg/m^2^) | 17.2 (16.9, 17.5) | 0.08 (-0.77, 0.93) | 16.9 (16.6, 17.3) | 1.08 (0.43, 1.73) | 17.2 (16.9, 17.5) | 1.02 (0.36, 1.67) |
| Δ 6 months – 2 years (kg/m^2^/week) | -0.01 (-0.02, -0.01) | 0.004 (-0.009, 0.01) | -0.01 (-0.01, -0.007) | -0.01 (-0.02, -0.002) | -0.01 (-0.02, -0.01) | -0.006 (-0.01, 0.004) |
| BMI at 2 years (kg/m^2^) | 15.9 (15.6, 16.2) | 0.39 (-0.45, 1.24) | 15.9 (15.6, 16.3) | 0.09 (-0.51, 0.7) | 16.02 (15.7, 16.3) | 0.54 (-0.13, 1.23) |
| Δ 2 years – 5 years (kg/m^2^/week) | 0.0002 (-0.001, 0.002) | -0.0002 (-0.006, 0.005) | 0.0004 (-0.002, 0.003) | -0.0005 (-0.004, 0.003) | 0.0001 (-0.001, 0.002) | -0.001 (-0.006, 0.002) |
| BMI at 5 years (kg/m^2^) | 15.9 (15.7, 16.2) | 0.36 (-0.35, 1.08) | 16.0 (15.7, 16.3) | 0.007 (-0.53, 0.54) | 16.0 (15.7, 16.3) | 0.25 (-0.37, 0.88) |
| Δ 5 years – 10 years (kg/m^2^/week) | 0.007 (0.005, 0.009) | 0.001 (-0.003, 0.006) | 0.007 (0.005, 0.009) | 0.00 (-0.003, 0.003) | 0.007 (0.005, 0.008) | 0.0004 (-0.003, 0.004) |
| BMI at 10 years (kg/m^2^) | 17.8 (17.4, 18.3) | 0.72 (-0.41, 1.86) | 17.9 (17.5, 18.4) | 0.005 (-0.83, 0.84) | 17.9 (17.5, 18.3) | 0.35 (-0.6, 1.31) |
| *Adjusted* |  |  |  |  |  |  |
| BMI at birth (kg/m^2^) | 13.9 (13.3, 14.4) | 1.61 (1.19, 2.03) | 13.4 (12.8, 13.9) | 1.17 (0.87, 1.48) | 14.4 (13.9, 14.8) | 2.15 (1.83, 2.46) |
| Δ Birth – 6 months (kg/m^2^/week) | 0.11 (0.05, 0.16) | -0.07 (-0.11, -0.03) | 0.11 (0.05, 0.17) | -0.003 (-0.03, 0.02) | 0.1 (0.04, 0.15) | -0.04 (-0.07, -0.01) |
| BMI at 6 months (kg/m^2^) | 16.7 (15.3, 18.2) | -0.28 (-1.2, 0.64) | 16.2 (14.8, 17.7) | 1.07 (0.37, 1.77) | 17.0 (15,6, 18.3) | 1.02 (0.28, 1.76) |
| Δ 6 months – 2 years (kg/m^2^/week) | -0.007 (-0.03, 0.01) | 0.009 (-0.005, 0.02) | -0.003 (-0.02, 0.02) | -0.01 (-0.02, -0.0001) | -0.005 (-0.02, 0.01) | -0.004 (-0.01, 0.006) |
| BMI at 2 years (kg/m^2^) | 16.1 (14.8, 17.5) | 0.47 (-0.42, 1.38) | 16.0 (14.6, 17.4) | 0.19 (-0.44, 0.84) | 16.5 (15.2, 17.9) | 0.64 (-0.09, 1.39) |
| Δ 2 years – 5 years (kg/m^2^/week) | -0.0001 (-0.009, 0.009) | -0.0007 (-0.007, 0.005) | 0.0008 (-0.008, 0.01) | -0.001 (-0.006, 0.002) | -0.001 (-0.01, 0.007) | -0.002 (-0.006, 0.002) |
| BMI at 5 years (kg/m^2^) | 16.1 (15.1, 17.1) | 0.36 (-0.39, 1.12) | 16.1 (15.1, 17.2) | -0.05 (-0.61, 0.5) | 16.3 (15.2, 17.4) | 0.28 (-0.4, 0.96) |
| Δ 5 years – 10 years (kg/m^2^/week) | 0.008 (0.002, 0.01) | 0.0003 (-0.004, 0.005) | 0.008 (0.002, 0.01) | -0.0007 (-0.004, 0.002) | 0.007 (0.002, 0.01) | -0.0002 (-0.003, 0.003) |
| BMI at 10 years (kg/m^2^) | 18.3 (16.9, 19.8) | 0.45 (-0.65, 1.56) | 18.4 (16.9, 19.8) | -0.25 (-1.04, 0.53) | 18.4 (16.9, 19.9) | 0.2 (-0.74, 1.15) |
| Abbreviations: BMI Body mass index; CI Confidence interval; Δ the change. Adjusted models controlled for original study group, child sex, HP index, maternal age at delivery, maternal ethnicity, gestational weight gain, maternal smoking in pregnancy, maternal physical activity in pregnancy, adherence to a special diet in pregnancy, metabolic complications in pregnancy, and paternal BMI. Mean trajectory is centred on the mean of the first measurement (birth) as the reference category. N = 331 | | | | | | |

| Supplementary Table 9: Sensitivity analyses of adjusted mean trajectory and mean difference in trajectory of anthropometry from birth to 10 years of age for birthweight ≥4 kg additionally adjusting for breastfeeding, estimated from multilevel linear spline models. | | |
| --- | --- | --- |
|  | **Mean weight trajectory (95% CI) (kg) for those born <4 kg** | **Difference (95% CI) in mean weight trajectory for those born ≥4 kg** |
| Weight at birth (kg) | 3.67 (3.49, 3.85) | 0.49 (0.41, 0.57) |
| Δ Birth – 6 months (kg/week) | 0.12 (0.07, 0.17) | 0.005 (-0.01, 0.02) |
| Weight at 6 months (kg) | 6.91 (5.61, 8.22) | 0.64 (0.15, 1.14) |
| Δ 6 months – 2 years (kg/week) | 0.06 (0.04, 0.08) | -0.0002 (-0.008, 0.007) |
| Weight at 2 years (kg) | 11.8 (10.3, 13.4) | 0.63 (-0.02, 1.28) |
| Δ 2 years – 5 years (kg/week) | 0.04 (0.03, 0.05) | -0.001 (-0.004, 0.002) |
| Weight at 5 years (kg) | 19.2 (17.5, 20.8) | 0.47 (-0.26, 1.2) |
| Δ 5 years – 10 years (kg/week) | 0.06 (0.05, 0.08) | -0.003 (-0.009, 0.002) |
| Weight at 10 years (kg) | 36.3 (32.4, 40.3) | -0.41 (-2.2, 1.37) |
|  | **Mean length/height trajectory (95% CI) (cm) for those born <4 kg** | **Difference (95% CI) in mean length/height trajectory for those born ≥4 kg** |
| Length at birth (cm) | 51.7 (50.6, 52.8) | 0.92 (0.42, 1.42) |
| Δ Birth – 6 months (cm/week) | 0.6 (0.51, 0.7) | -0.006 (-0.04, 0.02) |
| Length at 6 months (cm) | 67.5 (65.1, 70.1) | 0.76 (-0.18, 1.71) |
| Δ 6 months – 2 years (cm/week) | 0.23 (0.19, 0.27) | -0.002 (-0.01, 0.01) |
| Height at 2 years (cm) | 85.9 (83.4, 88.4) | 0.53 (-0.49, 1.56) |
| Δ 2 years – 5 years (cm/week) | 0.15 (0.13, 0.16) | -0.002 (-0.009, 0.005) |
| Height at 5 years (cm) | 109.4 (106.7, 112.2) | 0.21 (-1.02, 1.46) |
| Δ 5 years – 10 years (cm/week) | 0.12 (0.11, 0.13) | -0.0004 (-0.004, 0.003) |
| Height at 10 years (cm) | 141.2 (137.9, 144.4) | 0.09 (-1.38, 1.57) |
|  | **Mean BMI trajectory (95% CI) (kg/m^2^) for those born <4 kg** | **Difference (95% CI) in mean BMI trajectory for those born ≥4 kg** |
| BMI at birth (kg/m^2^) | 13.5 (12.6, 14.4) | 1.37 (0.97, 1.78) |
| Δ Birth – 6 months (kg/m^2^/week) | 0.06 (-0.01, 0.15) | -0.02 (-0.05, 0.006) |
| BMI at 6 months (kg/m^2^) | 15.3 (13.3, 17.2) | 0.68 (-0.05, 1.42) |
| Δ 6 months – 2 years (kg/m^2^/week) | 0.003 (-0.02, 0.03) | 0.001 (-0.009, 0.01) |
| BMI at 2 years (kg/m^2^) | 15.5 (13.8, 17.2) | 0.81 (0.11, 1.52) |
| Δ 2 years – 5 years (kg/m^2^/week) | 0.002 (-0.009, 0.01) | -0.003 (-0.008, 0.001) |
| BMI at 5 years (kg/m^2^) | 15.9 (14.5, 17.2) | 0.25 (-0.35, 0.86) |
| Δ 5 years – 10 years (kg/m^2^/week) | 0.007 (0.0005, 0.01) | -0.001 (-0.004, 0.001) |
| BMI at 10 years (kg/m^2^) | 17.8 (16.1, 19.5) | -0.1 (-0.86, 0.65) |
|  | **Mean WC (95% CI) (cm) for those born <4 kg** | **Difference (95% CI) in mean WC trajectory for those born ≥4 kg** |
| WC at birth (cm) | 34.9 (32.3, 37.5) | 2.08 (0.71, 3.45) |
| Δ Birth – 6 months (cm/week) | 0.26 (0.1, 0.43) | -0.02 (-0.1, 0.04) |
| WC at 6 months (cm) | 41.9 (38.4, 45.4) | 1.35 (0.04, 2.67) |
| Δ 6 months – 2 years (cm/week) | 0.08 (0.03, 0.14) | -0.01 (-0.03, 0.01) |
| WC at 2 years (cm) | 48.7 (45.7, 51.7) | 0.56 (-0.61, 1.75) |
| Δ 2 years – 5 years (cm/week) | 0.03 (0.01, 0.06) | -0.002 (-0.01, 0.006) |
| WC at 5 years (cm) | 54.7 (52.1, 57.2) | 0.18 (-0.99, 1.36) |
| Δ 5 years – 10 years (cm/week) | 0.03 (0.01, 0.05) | -0.004 (-0.01, 0.004) |
| WC at 10 years (cm) | 64.2 (58.8, 69.7) | -1.1 (-3.56, 1.35) |
| Abbreviations: BMI Body mass index; CI Confidence interval; Δ the change. Adjusted models controlled for original study group, child sex, HP index, maternal age at delivery, maternal ethnicity, gestational weight gain, maternal smoking in pregnancy, maternal physical activity in pregnancy, adherence to a special diet in pregnancy, metabolic complications in pregnancy, paternal BMI and breastfeeding. Mean trajectory is centred on the mean of the first measurement (birth) as the reference category. N = 183 | | |
